# Heart failure: mechanistic insights and precision therapeutic strategies

**DOI:** 10.3389/fcvm.2025.1696793

**Published:** 2025-12-05

**Authors:** Fang Zhang, Xiang Zhang, Jing Jian, Xu Zeng, Chao Zheng, Yaxi Zhang, Jinquan Gao

**Affiliations:** Department of Cardiology, Chongzhou People’s Hospital, Chongzhou, Sichuan, China

**Keywords:** heart failure, molecular mechanisms, fibrosis, mitochondrial dysfunction, inflammation, precision medicine

## Abstract

Heart failure (HF) is a major global health problem associated with high illness rates, mortality, and healthcare costs. Although advances in diagnosis and therapy have improved outcomes for some patients, effective treatment—especially for HF with preserved ejection fraction (HFpEF)—remains limited. HF develops through complex interactions among neurohormonal activation, metabolic remodeling, mitochondrial dysfunction, inflammation, fibrosis, and microvascular impairment. Recent discoveries in these areas have revealed new molecular and cellular targets that may lead to more precise therapies. Novel pharmacological agents, metabolic modulators, device-based interventions, and regenerative approaches are reshaping the treatment landscape. In addition, personalized strategies such as multi-omics profiling, biomarker-guided management, and artificial intelligence–assisted diagnosis hold promise for better risk prediction and individualized care. However, translating mechanistic discoveries into clinical benefit remains a challenge. Future research integrating molecular insights with clinical phenotyping will be essential to achieve precision treatment and improved outcomes in patients with HF.

## 1 Introduction

Heart failure (HF) is a complex clinical syndrome characterized by impaired ventricular filling or ejection due to structural or functional cardiac abnormalities. It leads to symptoms such as dyspnea, fatigue, and edema, and is associated with poor quality of life and prognosis (1). Based on left ventricular ejection fraction (LVEF), HF is classified into HF with reduced ejection fraction (HFrEF, LVEF <40%), mildly reduced ejection fraction (HFmrEF, 40%–49%), and preserved ejection fraction (HFpEF, ≥50%) (2, 3). Notably, HFpEF now accounts for more than half of all HF cases and represents a major therapeutic challenge (4).

Despite significant progress in pharmacological and device-based therapies, the overall prognosis of HF remains poor, with high rates of hospitalization and mortality (5). While neurohormonal inhibition with ACE inhibitors, β-blockers, and mineralocorticoid receptor antagonists has greatly improved outcomes in HFrEF, similar success has not been achieved in HFpEF (6). No current treatment convincingly reduces mortality in HFpEF, largely because of its heterogeneous mechanisms involving metabolic abnormalities, inflammation, microvascular dysfunction (MVD), and systemic comorbidities (7). These limitations highlight an urgent unmet clinical need for new, mechanism-based and precision-oriented therapeutic strategies.

This review therefore summarizes recent advances in understanding the molecular and cellular mechanisms of HF and discusses how these insights can inform novel pharmacological, device-based, and personalized treatment approaches aimed at improving outcomes across diverse HF phenotypes.

## 2 Methodological framework

This narrative review was conducted to synthesize recent advances in the mechanistic understanding and emerging therapeutic strategies of HF. A structured literature search was performed across the PubMed, Scopus, and Web of Science databases for studies published between January 2010 and September 2025. The search combined the following keywords and Boolean operators: “heart failure” AND (“molecular mechanism” OR “pathophysiology” OR “metabolic remodeling” OR “mitochondrial dysfunction” OR “fibrosis” OR “epigenetic regulation” OR “precision medicine” OR “therapeutic strategies”). Inclusion criteria were: (1) peer-reviewed original articles or reviews; (2) studies involving human subjects, relevant animal models, or translational data; and (3) publications in English. Exclusion criteria included case reports, editorials, conference abstracts, and non-peer-reviewed materials (8).

Reference lists of relevant articles and recent high-impact reviews were also screened to ensure comprehensive coverage. The quality and relevance of studies were assessed based on methodological rigor and contribution to mechanistic or therapeutic understanding. While this review is primarily narrative in scope, it follows key principles of the PRISMA-ScR framework, ensuring transparent reporting of literature identification and selection. All included references were cross-verified for accuracy and represent the most recent and impactful studies available at the time of writing (9).

## 3 Novel insights into the pathophysiological mechanisms of HF

**Figure 1:**
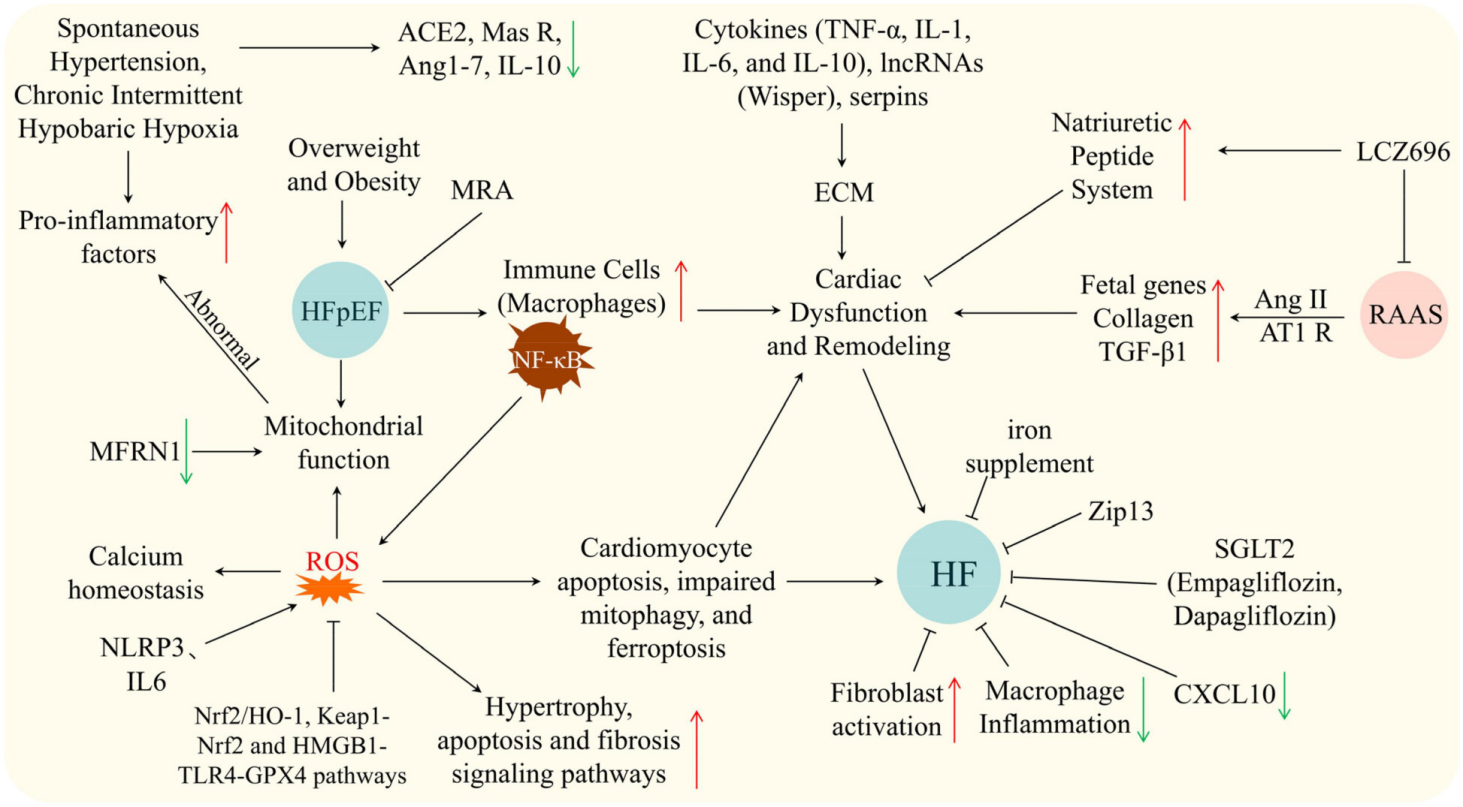
Schematic showing mechanisms and therapies in HF/HFpEF: triggers (hypertension, hypoxia, obesity), pathways (inflammation, ROS, RAAS, cytokines), cellular events, and interventions. Arrows: ↑ upregulation, ↓ downregulation, black for causal relationships, ⊥ for inhibition.

The pathophysiology of HF is highly complex, involving multiple mechanisms such as neuroendocrine activation, metabolic remodeling, inflammatory responses, mitochondrial dysfunction, and cellular senescence (10). These interacting processes, summarized in Figure 1, highlight the multi-layered nature of HF progression. In recent years, advances in experimental techniques have progressively elucidated more specific molecular mechanisms, providing a theoretical foundation for precision therapy.

### 3.1 Experimental insights into RAAS-mediated neuroendocrine activation in HF

Overactivation of the renin–angiotensin–aldosterone system (RAAS) and the sympathetic nervous system (SNS) is a major driving factor in the onset and progression of HF (11, 12). Under pathological conditions such as myocardial injury and hypertension, the key RAAS effector molecule angiotensin II (Ang II) mediates signal transduction via the AT1A receptor, upregulating the expression of fetal genes, collagen, and TGF-β1 in non-infarcted regions, thereby promoting left ventricular dilation, fibrosis, and dysfunction, and accelerating ventricular remodeling and HF progression (13). Multiple animal studies have confirmed the cardioprotective effects of RAAS inhibition on cardiac structure and function. For instance, Woźniak et al. (14) demonstrated in a Tgaq*44 dilated cardiomyopathy mouse model that early combined administration of an ACE inhibitor (perindopril) and an aldosterone receptor antagonist (canrenone) preserved systolic function, whereas late intervention mainly attenuated ventricular dilation, indicating a stage-dependent therapeutic effect. Chen et al. (15) reported in spontaneously hypertensive rats that chronic intermittent hypobaric hypoxia downregulated ACE and AT1 receptor expression, upregulated ACE2 and Mas receptor expression, reduced Ang II and pro-inflammatory cytokines, and increased Ang1–7 and the anti-inflammatory cytokine IL-10, thereby improving vascular relaxation and remodeling. Hawlitschek et al. (16) found that captopril alone or in combination with nifedipine significantly reduced blood pressure, alleviated cardiac hypertrophy, and prevented myocardial fibrosis. Yi et al. (17) showed that time-restricted feeding suppressed the ACE–Ang II–AT1 axis, reducing Ang II-mediated cardiac remodeling and dysfunction, thereby lowering blood pressure and exerting cardioprotective effects. Clinically, McMurray et al. (18) demonstrated in the PARADIGM-HF trial that the angiotensin receptor–neprilysin inhibitor (ARNI) LCZ696 not only inhibited RAAS activity but also enhanced natriuretic peptide system function, synergistically improving cardiac load and neurohormonal imbalance, and producing greater reductions in cardiovascular mortality and HF rehospitalization compared with the ACE inhibitor enalapril. Collectively, these findings underscore the central pathological role of RAAS in cardiovascular disease progression, and indicate that RAAS inhibition yields significant improvements in cardiac structure and function in experimental models, with clear prognostic benefits in clinical practice.

### 3.2 Exercise and metabolic modulation in HF

Metabolic abnormalities are a key component of HF pathophysiology. Overweight and obesity, even in the absence of metabolic syndrome, significantly increase HF risk (by 37%–85%), with cardiovascular event risk rising proportionally with the number of metabolic syndrome components (19). In HFpEF, epicardial adipose tissue volume is markedly increased and correlates with cardiac dysfunction, metabolic abnormalities, and inflammatory markers (20).

Pharmacologically, mineralocorticoid receptor antagonists (MRAs) reduce HF hospitalization and cardiovascular mortality in HFrEF, with more modest benefits in HFmrEF/HFpEF and an increased risk of hyperkalemia (21). Non-steroidal MRA finerenone significantly reduces HF composite outcomes in patients with recent worsening HF (WHF) without increasing adverse events (22), while finerenone also lowers the risk of new-onset diabetes by 24% in HF patients without baseline diabetes (23). Among SGLT2 inhibitors, dapagliflozin achieves similar diuretic efficacy to metolazone but with less electrolyte disturbance and renal function deterioration (24), whereas empagliflozin shows no short-term improvement in myocardial energy metabolism (25). The mitochondrial uncoupler HU6 may enhance metabolic flexibility and potentially improve cardiac function in obesity-related HFpEF (26).

Exercise interventions show heterogeneous effects. In HFpEF, high-intensity interval training (HIIT) does not outperform moderate-intensity continuous training (MICT) in improving peak oxygen uptake (VO_2_peak) (27). However, in coronary artery disease and some chronic HF populations, HIIT significantly improves VO_2_peak, heart rate variability, and left ventricular function (28–30). Cardiac rehabilitation (CR) reduces HF and all-cause rehospitalization and improves exercise capacity and quality of life, with no mortality benefit (31), while hybrid comprehensive telerehabilitation (HCTR) yields short-term functional gains without long-term clinical benefit (32). Additionally, exercise oscillatory ventilation independently predicts mortality and heart transplantation in HFrEF (33). Other interventions, such as oral polysaccharide iron in iron-deficient HFrEF, do not improve exercise capacity or cardiac function (34). Collectively, these findings highlight the need for integrated metabolic control, optimized pharmacotherapy, and individualized exercise prescriptions to maximize benefits in HF management.

### 3.3 Mitochondrial dysfunction and iron metabolism in HF

Accumulating evidence from basic and clinical studies indicates that mitochondrial dysfunction is a central mechanism in the development and progression of HF. Impaired mitochondrial respiration in cardiomyocytes and peripheral blood mononuclear cells (PBMCs) exacerbates systemic inflammation. Zhou et al. (35) demonstrated that MitoDAMPs suppress complex I activity in PBMCs via IL-6 induction, whereas supplementation with the NAD^+^ precursor nicotinamide riboside (NR) enhances mitochondrial respiration and attenuates pro-inflammatory cytokine expression. Disruption of mitochondrial iron homeostasis also contributes critically to HF pathophysiology. Li et al. (36) reported that cardiac-specific Zip13 knockout mice exhibit severe contractile dysfunction, with decreased mitochondrial iron and elevated cytosolic iron; iron supplementation or overexpression of the mitochondrial iron transporter MFRN1 partially restores mitochondrial function, highlighting the essential role of ZIP13 in maintaining myocardial mitochondrial iron balance. Clinically, approximately 23% of HF patients present with left ventricular myocardial iron deficiency, which correlates with reduced activity of mitochondrial respiratory chain complexes and TCA cycle enzymes, as well as impaired oxidative stress defense (37). *In vitro* iron deprivation further confirms that impaired Fe–S cluster-dependent complex I–III activity significantly inhibits ATP production and cardiomyocyte contractility, which can be reversed by iron supplementation (38). Furthermore, frataxin deficiency or SLC25A3 loss perturbs NAD^+^ metabolism, mitochondrial biogenesis, and fusion/fission dynamics, leading to metabolic dysfunction, Ca^2+^ handling abnormalities, and cardiomyocyte hypertrophy (39, 40), while mitochondrial transplantation partially rescues these phenotypes. In endothelial cells, lipid droplet formation mitigates lipotoxicity, preserves mitochondrial function, and inhibits ferroptosis, thereby protecting cardiac microvascular integrity (41). Clinically, intravenous iron administration or SGLT2 inhibition with empagliflozin improves mitochondrial energy metabolism, enhances iron utilization and erythropoiesis, and subsequently improves cardiac and skeletal muscle function, leading to enhanced exercise capacity and left ventricular performance in HF patients (42–45). Collectively, mitochondrial dysfunction in HF involves deficits in energy metabolism as well as dysregulation of iron homeostasis and NAD^+^ levels, and targeting mitochondrial pathways represents a promising therapeutic strategy to improve cardiac function.

### 3.4 Inflammation in HF

A growing body of epidemiological and clinical evidence demonstrates that inflammation plays a central role in the onset, progression, and prognosis of HF, particularly HFpEF. Elevated systemic immune-inflammation index (SII) is positively associated with HF risk in multiple populations, including NHANES cohorts, smokers, and patients with diabetes (46, 47). Chronic immune-inflammatory conditions, such as rheumatoid arthritis, increase the risk of both ischemic and non-ischemic HF, with the highest risk observed in rheumatoid factor–positive individuals (48). HFpEF is frequently accompanied by metabolic abnormalities and activation of systemic inflammatory protein networks, in which specific mediators—TNFR1, UPAR, IGFBP7, and GDF-15—partially mediate structural and functional cardiac impairment (49), while distinct inflammatory patterns are associated with adverse outcomes, reduced exercise capacity, and impaired quality of life (50). These findings support the existence of a “comorbidity–inflammation–HF” pathological axis, in line with mechanistic insights that metabolic derangements and inflammatory burden interact to drive metabolic inflammation (metainflammation) (51), promoting ventricular remodeling through immune cell polarization, pro-inflammatory cytokine release, oxidative stress, and pathological fibrosis. Experimental studies further demonstrate that HF is associated with pro-inflammatory activation of immune cells (e.g., macrophages), mitochondrial dysfunction, and activation of key signaling pathways such as NF-κB; inhibiting macrophage inflammation, promoting M2 polarization, or restoring mitochondrial function can attenuate cardiac dysfunction and remodeling (35, 52). In terms of anti-inflammatory interventions, sodium-glucose cotransporter 2 (SGLT2) inhibitors such as empagliflozin and dapagliflozin exhibit significant cardioprotective effects in HF models and patients (53, 54), partly independent of SGLT2 itself, involving downregulation of CXCL10 (55), suppression of macrophage inflammation, and modulation of fibroblast activation. Additional approaches—including low-level transcutaneous vagus nerve stimulation (56, 57), selenium-enriched diets (58), NAD precursors (35), NF-κB pathway inhibitors, and certain herbal preparations such as processed Aconitum extracts (59)—have also demonstrated potential to improve cardiac function via inflammation attenuation. However, previous trials of anti-inflammatory agents such as TNF-α antagonists have shown limited or even harmful effects in HF (48), suggesting that non-specific or isolated anti-inflammatory strategies may be insufficient. Collectively, these findings underscore that although inflammation is an indispensable mechanistic link in HF, the efficacy of anti-inflammatory therapy depends on pathway selectivity, timing of intervention, and alignment with patient phenotype, rather than broad cytokine inhibition. Future therapies may benefit from biomarker-guided and phenotype-specific strategies that better match the underlying inflammatory pathways in HF.

### 3.5 Oxidative stress and cardiomyocyte injury in HF

In HF, the high metabolic activity of cardiomyocytes renders them a primary source of reactive oxygen and nitrogen species (ROS/RNS), while neurohormonal activation, adrenergic overstimulation, and excessive mechanical stress induce cellular stress that disrupts redox homeostasis and impairs mitochondrial function (60, 61). Major ROS sources include mitochondrial electron transport chain complexes I/III, NADPH oxidases (Nox2/Nox4), xanthine oxidoreductase (XOR), nitric oxide synthases (NOS), monoamine oxidases (MAO), and p66shc. Enhanced activity of these enzymes establishes a ROS-driven vicious cycle, damaging mitochondrial DNA, proteins, and lipids, perturbing calcium homeostasis, and activating hypertrophic, apoptotic, and fibrotic signaling pathways (62–64). Excess ROS can further induce cardiomyocyte apoptosis, impaired mitophagy, and ferroptosis through IGF2BP2-dynamin2, PDE4D-CREB-SIRT1-PINK1/Parkin, and Piezo1/Yap1 signaling axes, accelerating myocardial remodeling and HF progression (65–67). Activation of the NLRP3 inflammasome also exacerbates cardiomyocyte injury by promoting oxidative stress and pyroptosis (68). Under pathological conditions, excessive ROS generation combined with impaired antioxidant defense systems, including SOD, GSHPx, catalase, Trx/TrxR, and GSH, leads to cumulative oxidative damage, further compromising cardiomyocyte structure and function (62). Therapeutic strategies activating Nrf2/HO-1, Keap1-Nrf2, and HMGB1-TLR4-GPX4 pathways can suppress ROS production, restore mitochondrial function, and mitigate cardiomyocyte apoptosis, inflammation, and fibrosis, thereby improving pathological cardiac remodeling (69–71). Collectively, ROS generation in cardiomyocytes and its dynamic regulation constitute a central driver of myocardial remodeling and HF progression, providing a mechanistic basis for targeted interventions against cardiomyocyte oxidative stress (72).

### 3.6 Fibrosis and extracellular matrix remodeling in HF

Myocardial fibrosis is a central pathological process in the development and progression of HF, characterized by the activation of cardiac fibroblasts (CFs) and their differentiation into myofibroblasts (myoFbs), which mediate excessive deposition and crosslinking of extracellular matrix (ECM) proteins, ultimately leading to ventricular stiffening, myocardial remodeling, and functional impairment (73–75). Fibrosis can represent either a reparative response or maladaptive remodeling: acute myocardial injury, such as myocardial infarction, induces replacement fibrosis to preserve myocardial structural integrity, whereas chronic pressure overload triggers reactive fibrosis, resulting in diffuse interstitial and perivascular collagen accumulation and persistent disruption of left and right ventricular function (76, 77).

CFs sense mechanical stress and integrate signaling via integrin-FAK pathways, AngII downstream cascades, and TGF-β/Smad and non-canonical MAPK/PI3K/AKT pathways to regulate proliferation, migration, and phenotypic transformation, thereby driving excessive deposition of type I/III collagen and other matrix proteins (73, 74, 78, 79). Additionally, immune cell-mediated inflammation, cytokines (including TNF-α, IL-1, IL-6, IL-10), long noncoding RNAs such as Wisper, fibronectin, and serpins participate in myocardial ECM remodeling by modulating fibroblast activity, collagen crosslinking, and matrix degradation, further influencing fibrotic progression (80–83).

Myocardial fibrosis not only reduces diastolic compliance and impairs systolic function but also disrupts intercellular signaling and increases the risk of arrhythmias (84, 85). Interventions such as left ventricular assist device (LVAD) unloading, HDAC inhibitors, SGLT2 inhibitors, and direct fibroblast reprogramming have been shown to partially reverse fibrosis and structural-functional abnormalities, highlighting its potential as a therapeutic target in HF (75, 86–88). Collectively, myocardial fibrosis, through ECM remodeling, plays a pivotal role in HF pathogenesis, and the complex molecular regulatory networks and cellular heterogeneity underlying this process provide important directions for future precision therapies (89–91).

### 3.7 Autophagy and proteostasis dysregulation in HF

Autophagic dysregulation and protein homeostasis imbalance play central roles in the pathogenesis and progression of HF. Autophagy maintains cardiomyocyte protein quality control (PQC), clears misfolded and toxic proteins, and preserves mitochondrial function, with moderate activation conferring cardioprotection (92, 93). However, excessive or insufficient autophagy disrupts protein homeostasis, induces mitochondrial damage, and triggers apoptosis or autophagy-dependent cell death (autosis), exacerbating cardiomyopathy, atrial fibrillation, and HF progression (94, 95). PKG signaling and PQC dysfunction accelerate hypertrophy, remodeling, and cardiomyocyte loss, whereas pharmacological PKG activation restores PQC and improves cardiac function (96). BAG3 maintains protein homeostasis via chaperone-assisted selective autophagy (CASA), and its deficiency leads to age-dependent autophagic dysregulation and early-onset cardiomyopathy (97).

In HFpEF, diabetes promotes NF-κB/IL-6/NLRP3-mediated inflammation and oxidative stress, suppresses NO-sGC-cGMP-PKG and AKT/AMPK/mTOR-regulated autophagy, and impairs HSP27/HSP70-dependent PQC, increasing myocardial stiffness and diastolic dysfunction (98). Nrf2 regulates the ubiquitin–proteasome system and autophagy-related genes, and its dysfunction drives protein misfolding, hypertrophy, and HF development (99). Persistent mTORC1/4EBP1 activation under aging or metabolic–hypertensive stress disrupts protein homeostasis, accelerates toxic protein accumulation, and exacerbates HFpEF and cardiac aging (100, 101). Additional regulators, including ROR2, TRIM24, and Txlnb, modulate protein folding, ubiquitination, and calcium handling, further destabilizing protein homeostasis and impairing contractile function (102–105). Models of chronic kidney disease, premature aging, and rapid-growth broiler cardiomyopathy demonstrate that protein oxidation, glycation, and PQC disruption, combined with autophagic and mitochondrial dysfunction, accelerate pathological remodeling (106–108).

Recent studies have further delineated the crosstalk between autophagy, mitochondrial health, and proteostasis. Dysregulated mitophagy contributes to the accumulation of dysfunctional mitochondria and excessive ROS generation, amplifying cardiomyocyte injury and maladaptive remodeling (109). Crosstalk between mTOR, AMPK, and sirtuin signaling fine-tunes autophagic flux, linking nutrient status, metabolic stress, and cellular aging to HF progression (110). Moreover, key autophagy mediators such as Beclin-1, ATG5, and TFEB are increasingly recognized as potential therapeutic targets because restoration of balanced autophagy—neither excessive nor suppressed—attenuates hypertrophy, fibrosis, and contractile dysfunction in preclinical models (111). These findings reinforce the translational potential of modulating the AMPK–mTOR–TFEB axis or enhancing mitophagy to improve proteostasis and delay HF progression.

Therapeutically, interventions such as tanshinone IIA, rapamycin, ginsenosides, and components of Si-Miao-Yong-An decoction modulate AMPK–mTOR–Beclin1-dependent autophagy, inhibit mTORC1 and ER stress, or restore mitophagy, thereby reducing apoptosis, improving function, and attenuating remodeling (112–115). Nutritional status further influences autophagic activity, highlighting the interplay between autophagy and protein homeostasis as a potential therapeutic target in chronic HF (116). Collectively, autophagic dysregulation and protein homeostasis imbalance constitute central mechanisms driving cardiomyocyte injury, remodeling, and HF progression, providing avenues for precision therapy (117).

### 3.8 MVD and endothelial abnormalities in HF

Multiple studies have demonstrated that MVD and coronary microvascular endothelial dysfunction are highly prevalent and play critical pathogenic roles in HF, particularly in HFpEF. Even in HFpEF patients without obstructive coronary artery disease, approximately 81% exhibit coronary microvascular dysfunction (CMD), suggesting it as a potential therapeutic target (118). CMD can reduce coronary flow reserve and myocardial perfusion, thereby exacerbating myocardial ischemia, diastolic dysfunction, and oxidative stress, ultimately promoting HF progression and increasing cardiovascular risk (119). In HFpEF patients, coronary microvascular endothelial dysfunction involves both endothelium-dependent and endothelium-independent mechanisms, with the latter closely associated with impaired diastolic function and adverse outcomes (120, 121).

MVD impairs endothelial cell function, decreases nitric oxide (NO) bioavailability, and reduces coronary flow reserve, promoting left ventricular diastolic dysfunction and restrictive remodeling while compromising myocardial oxygen supply and preload reserve through multiple interacting pathways (122). Peripheral microvascular reactivity is also correlated with left ventricular structural and diastolic alterations, highlighting its role in early cardiac remodeling in HFpEF (123). Endothelial dysfunction in HF patients manifests as impaired vascular tone regulation, antioxidant capacity, and inflammatory modulation, which may be systemic or localized, and can serve as a prognostic marker while being partially modifiable by ACE inhibitors, statins, and regular exercise (124). Clinical studies further indicate that CMD restricts cardiac filling during exercise, reduces exercise capacity, and is closely linked to left ventricular diastolic dysfunction and myocardial ischemia (120). Additionally, HFpEF patients often present with arterial stiffening, impaired microvascular vasodilation, and abnormal venous capacitance, which collectively contribute to a complex pathophysiological network (125).

In summary, MVD and endothelial impairment constitute key pathogenic mechanisms in HF and HFpEF, promoting disease progression through myocardial ischemia, diastolic dysfunction, and adverse cardiac remodeling, while representing critical targets for early diagnosis, risk stratification, and individualized therapeutic intervention.

### 3.9 Epigenetic and transcriptomic regulation in HF

HF, particularly as a consequence of dilated cardiomyopathy (DCM), is intricately regulated by epigenetic and transcriptomic mechanisms. CFs, in response to injury, transition from a quiescent state to a highly collagen-secreting phenotype, thereby driving fibrosis and cardiac remodeling through excessive ECM deposition. This activation is precisely controlled by gene transcription, DNA methylation, histone modifications, chromatin remodeling, and intercellular signaling networks (126). Non-coding RNAs, including the lncRNA Wisper, interact with TIA1-related proteins to modulate pro-fibrotic enzyme expression, promoting fibrosis and adverse remodeling post-myocardial infarction, whereas antisense oligonucleotide-mediated silencing significantly alleviates fibrosis and cardiac dysfunction (83). Similarly, NAT10-mediated N4-acetylcytidine (ac4C) modification of mRNAs enhances the stability and translation efficiency of CD47 and ROCK2 transcripts, thereby facilitating cardiomyocyte hypertrophy, fibrosis, and inflammatory responses; inhibition of NAT10 or treatment with Remodelin effectively improves left ventricular structure and function (127). Epitranscriptomic enzymes and non-coding RNAs—including miRNAs, lncRNAs, and circRNAs—coordinate gene transcription and translation in cardiomyocytes and the cardiac immune microenvironment, influencing remodeling, diastolic dysfunction, inflammation, and fibrosis, and thus represent promising targets for diagnosis, prognosis, and therapeutic intervention in HF (128, 129).

Bromodomain and extra terminal domain (BET) family proteins, particularly BRD4, recognize histone acetylation marks and integrate super-enhancer and promoter regions of pro-fibrotic genes under cardiac stress, orchestrating NF-κB- and TGF-β-dependent fibrosis and contractile dysfunction while maintaining mitochondrial respiration and energy homeostasis under baseline conditions (130–132). Other epigenetic regulators, including DNA methyltransferases, protein methyltransferases, MLF1, HAND1, and Bmi1, modulate chromatin accessibility, enhancer-promoter looping, and mRNA splicing, thereby governing cardiomyocyte hypertrophy, fibrosis, calcium handling, and age-associated transcriptional programs (133–138). Environmental factors, such as bisphenol A and its analogs, can induce cardiomyocyte hypertrophy and alter DNA methylation patterns, illustrating the interplay between transcriptional and epigenetic mechanisms in cardiac toxicity (139). Multi-omics analyses reveal disease-specific DNA methylation and non-coding RNA expression patterns across HF patients of distinct etiologies, with epigenetic reprogramming and transcriptional dysregulation jointly dictating myocardial function, fibrosis, and remodeling (140, 141). Furthermore, stem cells and their derived cardiomyocytes, through optimized differentiation and maturation, can repair myocardial infarction-induced injury and improve cardiac function in HF patients (142).

Beyond individual epigenetic modifiers, emerging multi-omics studies have revealed coordinated epigenetic remodeling that regulates transcriptional plasticity in both cardiomyocytes and fibroblasts. Disease-specific DNA methylation signatures correlate with metabolic impairment and hypertrophy, whereas histone acetylation and m6A RNA modifications fine-tune gene networks that govern fibrosis, inflammation, and energy metabolism (143). Notably, pharmacological modulation of epigenetic pathways—such as inhibition of BRD4 or HDACs—has demonstrated the ability to reverse maladaptive gene expression, attenuate fibrosis, and improve ventricular function in preclinical models (144). These findings underscore epigenetic regulation as a mechanistically distinct yet clinically relevant avenue for precision therapy in both HFrEF and HFpEF (Table 1).

**Table 1:** Comparative summary of key mechanisms and therapeutic targets in HFrEF vs. HFpEF.

Pathophysiological domain	HFrEF (Reduced EF)	HFpEF (Preserved EF)	Representative or emerging therapies	References
Dominant Mechanism	Systolic dysfunction due to loss of contractile function and adverse remodeling	Diastolic dysfunction with preserved contractility but increased stiffness and impaired relaxation	——	(147)
Neurohormonal Activation (RAAS/SNS)	Markedly upregulated; drives remodeling and apoptosis	Mild to moderate activation; interacts with comorbid inflammation	ACEIs, ARBs, β-blockers, ARNIs	(148)
Metabolic Remodeling	Impaired fatty acid oxidation and ATP depletion	Metabolic inflexibility, obesity-related inflammation, insulin resistance	SGLT2 inhibitors, GLP-1 agonists, metabolic modulators	(149)
Mitochondrial Dysfunction & Oxidative Stress	Mitochondrial injury, excess ROS production	Mitochondrial inefficiency linked to systemic inflammation	NAD^+^ boosters, antioxidants, iron supplementation	(150)
Inflammation & Fibrosis	Driven by RAAS and mechanical stress	Chronic systemic and microvascular inflammation leading to stiffening	Anti-inflammatory agents, fibroblast reprogramming, SGLT2i	(151)
Autophagy & Proteostasis	Excessive or defective autophagy contributing to cell loss	Impaired autophagy with protein aggregation and metabolic stress	AMPK activators, mTOR inhibitors, PKG modulators	(152, 153)
Epigenetic Regulation	Chromatin remodeling and non-coding RNAs driving hypertrophy and fibrosis	Differential DNA methylation and transcriptomic reprogramming under metabolic stress	BET inhibitors, HDAC inhibitors, RNA therapeutics	(154)
MVD	Secondary to remodeling and ischemia	Primary driver causing impaired perfusion and diastolic dysfunction	Endothelial-targeted therapies, exercise, statins	(155)
Therapeutic Focus	Neurohormonal inhibition and device-based support	Metabolic and microvascular modulation; precision therapy	Combined mechanism-based and personalized approaches	(156)

Collectively, HF pathogenesis is orchestrated by multilayered networks encompassing non-coding RNA regulation, epigenetic enzymes, chromatin remodeling, and epitranscriptomic modifications, providing a comprehensive molecular framework for mechanistic understanding and potential precision therapeutic strategies (145, 146).

### 3.10 Evidence quality and limitations of current data

The strength of available evidence across HF mechanisms and therapies varies substantially. Mechanistic insights are often derived from preclinical or single-center translational studies, which provide valuable biological understanding but are limited by small sample sizes, short durations, and lack of clinical endpoints (157). Conversely, large RCTs—such as those evaluating RAAS inhibition, β-blockers, and SGLT2 inhibitors—offer high-level evidence but typically target broad HF populations without molecular stratification (158). Conflicting findings among studies frequently reflect differences in experimental design, patient phenotype, and outcome measures. Future research should aim to integrate mechanistic precision with rigorous clinical trial methodology to close this translational gap.

## 4 Therapeutic advances in HF

HF results from complex interactions among neurohormonal activation, metabolic dysregulation, inflammation, fibrosis, and vascular dysfunction. Understanding these mechanisms has guided the development of targeted therapies. The following sections summarize current and emerging strategies in HF, including pharmacological, device-based, and regenerative approaches.

### 4.1 Pharmacological and metabolic therapies in HF

HF is a complex cardiovascular syndrome with multifactorial pathophysiology, in which neurohormonal dysregulation plays a pivotal role. In HFrEF, chronic activation of the SNS and the RAAS initially maintains hemodynamic stability but ultimately exacerbates cardiac workload, promotes myocardial remodeling, and accelerates disease progression (159, 160). RAAS and SNS hyperactivity are also implicated in the pathogenesis of cardiorenal syndrome and pulmonary arterial hypertension-related right ventricular failure, highlighting the potential clinical value of neurohormonal inhibition (161, 162).

Evidence-based pharmacotherapy for HFrEF includes ACE inhibitors (ACEIs), angiotensin II receptor blockers (ARBs), β-adrenergic blockers, and MRAs. ACEIs and ARBs improve cardiovascular and renal outcomes by suppressing RAAS activity, whereas β-blockers attenuate β1-adrenergic overstimulation, reducing apoptosis, inflammation, and adverse remodeling; their effects on myocyte enhancer factor 2 (MEF2) signaling and downstream gene networks further contribute to improved left ventricular function and survival (163–165). A comprehensive overview of the mechanisms, clinical evidence, and limitations of these pharmacological strategies is provided in Table 2. Combination therapy strategies, such as ACEI/ARB plus β-blocker and MRA, or the use of ARNIs, which simultaneously block RAAS and enhance natriuretic peptide (NP) signaling, have demonstrated significant reductions in cardiovascular mortality and HF hospitalization, with favorable tolerability in clinical practice (166–170). Nevertheless, real-world utilization of these guideline-directed therapies remains suboptimal, and polypharmacy may increase the risk of renal impairment and hyperkalemia, underscoring the need for individualized dosing and combination strategies (171, 172).

**Table 2:** Pharmacologic therapies in HF: mechanisms, evidence, and limitations.

Drug class	Primary mechanism of action	Key clinical trial evidence	Major limitations/considerations	References
ACE Inhibitors/ARBs	Inhibit RAAS activation, reduce afterload and remodeling	SOLVD, VAL-HeFT, CHARM	Cough, hyperkalemia, renal dysfunction; limited efficacy in HFpEF	(181)
ARNIs (sacubitril/valsartan)	Dual RAAS blockade + neprilysin inhibition enhances natriuretic peptides	PARADIGM-HF, PARAGON-HF	Hypotension, renal monitoring required; cost higher than ACEI/ARB	(182)
β-Blockers	Inhibit sympathetic activation, reduce HR and remodeling	MERIT-HF, COPERNICUS, CIBIS-II	Contraindicated in bradycardia, acute decompensation	(183)
MRAs	Block aldosterone-induced fibrosis and Na^+^ retention	RALES, EMPHASIS-HF, TOPCAT	Risk of hyperkalemia, renal impairment	(184)
SGLT2 Inhibitors	Promote natriuresis, reduce preload/afterload, improve metabolism	DAPA-HF, EMPEROR-Reduced, DELIVER	Mild volume depletion, cost; broad efficacy across EF spectrum	(185)
Diuretics	Symptomatic relief via volume reduction	–	No mortality benefit; electrolyte imbalance	(186)
Ivabradine	Selective sinus node inhibitor lowering HR	SHIFT	Use only in sinus rhythm, HR >70 bpm	(187)
Vericiguat/Soluble Guanylate Cyclase Stimulators	Enhance NO-sGC-cGMP signaling, improve vascular function	VICTORIA	Limited to advanced HF; modest benefit	(188)
Omecamtiv Mecarbil	Direct myosin activator enhancing contractility	GALACTIC-HF	Requires LV systolic dysfunction; no mortality reduction	(189)
Anti-fibrotic/Anti-inflammatory agents (emerging)	Target TGF-β, NLRP3, IL-1 pathways	Ongoing early-phase trials	Preclinical/Phase II only; safety under evaluation	(190, 191)

For HFpEF and HFmrEF, conventional RAAS inhibitors and β-blockers show limited efficacy, and no definitive treatment exists (173–175). Recently, metabolic-targeted therapies, including sodium–glucose cotransporter 2 inhibitors (SGLT2i) and GLP-1 receptor agonists, have shown promise. SGLT2i exert cardiovascular benefits not only through renal glucose and sodium transport inhibition but also via modulation of cardiomyocyte ionic homeostasis, attenuation of inflammation and oxidative stress, thereby improving outcomes in both HFrEF and HFpEF patients (25, 176–178). GLP-1 receptor agonists ameliorate cardiac metabolic dysfunction, reduce myocardial hypertrophy and fibrosis, and improve cardiac function (179). Moreover, SGLT2i confer substantial cardiovascular benefits in elderly patients with type 2 diabetes mellitus, and metabolic interventions may also mitigate oxidative stress, neuroinflammation, and mitochondrial dysfunction, potentially improving cognitive decline, though clinical evidence remains limited (180).

Overall, HF pharmacotherapy is transitioning from simple suppression of maladaptive neurohormonal activation toward restoration of neuroendocrine balance and multi-targeted interventions. Future HF management may integrate conventional RAAS and β-blocker therapy with novel agents such as ARNIs, SGLT2i, and GLP-1 receptor agonists, along with metabolic interventions, to achieve cardiac protection, hemodynamic optimization, and metabolic homeostasis, providing a foundation for precision and individualized therapy (159).

### 4.2 Device-based and advanced interventional therapies in HF

HF patients continue to experience substantial residual risk despite guideline-directed medical therapy, including persistent symptoms, high rates of hospitalization, and mortality (192–194). These limitations have driven the rapid development of device-based interventions, which provide individualized therapies according to HF phenotype and severity. Key device therapies include cardiac resynchronization therapy (CRT), implantable cardioverter-defibrillators (ICD), mechanical circulatory support (MCS), and heart transplantation. An overview of these device-based and interventional approaches, including their indications, underlying mechanisms, and clinical limitations, is summarized in Table 3. CRT improves ventricular mechanical synchrony, enhancing left ventricular systolic function, reducing mitral regurgitation, promoting reverse remodeling, improving NYHA functional class and exercise capacity, and decreasing hospitalization and mortality (195–197). ICDs are primarily indicated for patients with moderate to severe HF or those at high risk, effectively preventing sudden cardiac death and ventricular arrhythmias, with outcomes influenced by NYHA class and cardiac function (198). For end-stage HF, MCS and heart transplantation provide definitive interventions, improving survival and quality of life, although device-related complications and limited availability remain challenges.

**Table 3:** Device-based and interventional therapies in HF.

Therapy type	Mechanistic principle	Key trials/evidence	Patient selection criteria	Limitations/cost considerations	References
CRT	Improves ventricular synchrony via biventricular pacing	COMPANION, CARE-HF	LVEF ≤35%, QRS ≥130 ms, sinus rhythm	Non-responders (∼30%); device cost	(207)
ICD	Prevents sudden cardiac death by terminating arrhythmias	MADIT-II, SCD-HeFT	Ischemic/non-ischemic cardiomyopathy, LVEF ≤35%	Shocks, infection, cost	(208)
LVAD	Provides mechanical circulatory support in advanced HF	REMATCH, MOMENTUM-3	End-stage HF awaiting or ineligible for transplant	Bleeding, infection, high cost	(209)
Baroreflex Activation Therapy	Modulates autonomic balance via carotid stimulation	BeAT-HF	Symptomatic HFrEF despite optimal therapy	Limited long-term data	(210)
CCM	Enhances myocardial calcium handling	FIX-HF-5C	LVEF 25%–45%, narrow QRS	Small population, cost	(211)
Interatrial Shunt Devices	Reduces left atrial pressure in HFpEF	REDUCE LAP-HF II	HFpEF with elevated LV filling pressure	Mixed results, long-term benefit uncertain	(212)
Transcatheter Mitral Valve Repair (TEER/MitraClip)	Corrects functional MR, reduces volume overload	COAPT, MITRA-FR	HF with secondary MR, suitable anatomy	Procedural risk, operator experience dependent	(213)

Clinical studies indicate that approximately one-third of CRT recipients are non-responders, with key contributing factors including suboptimal atrioventricular (AV) and interventricular (VV) timing, non-left bundle branch block (non-LBBB), and electromechanical dyssynchrony (199, 200). To address this, emerging device-based sensor technologies and automated optimization algorithms have shown promise in improving long-term clinical outcomes and reducing HF-related rehospitalization, with some evidence suggesting superiority over conventional echocardiography-guided optimization (201, 202). Moreover, concomitant atrial fibrillation (AF) may attenuate CRT-mediated improvements in left ventricular function, and AF burden should be considered in therapeutic strategy and device selection (203). Cardiac contractility modulation (CCM) improves left ventricular systolic function and promotes reverse remodeling in patients with mild QRS prolongation, achieving effects comparable to CRT in this subgroup, though CRT remains more effective in patients with severe QRS prolongation (204).

In elderly HF patients (≥75 years), CRT-D therapy has been shown to reduce HF progression, mortality, and the risk of ventricular arrhythmias, with device reintervention rates comparable to younger populations. However, older patients are at higher risk of device-related complications, and current clinical trials provide limited evidence regarding quality of life and end-of-life care considerations in this population (205, 206). Real-world data also indicate that HF functional class significantly impacts device outcomes: in patients receiving ICD alone, NYHA class III/IV patients exhibit higher risk of HF-related events or death compared with class I/II, whereas CRT-D attenuates this disparity; additionally, patients with milder HF are more prone to ventricular arrhythmias (198).

In summary, device-based therapies play a pivotal role in HF management by improving ventricular synchrony, preventing sudden cardiac death, and providing advanced circulatory support. Integration of individualized indications, outcome evaluation, and emerging device technologies—including automated optimization algorithms, minimally invasive lead placement, and sensor-based monitoring—offers precise and personalized interventions, highlighting the potential of these strategies to reduce residual risk and optimize clinical outcomes across diverse HF populations.

### 4.3 Emerging biological and personalized therapies in HF

HF is a complex syndrome involving interactions among cardiomyocytes, fibroblasts, immune cells, and vascular endothelial cells. Myocardial fibrosis plays a central role in HF progression, and its extent and pattern are closely associated with disease development and prognosis, which can be assessed via imaging and circulating biomarkers (214). Traditional biomarkers such as BNP and NT-proBNP remain essential indicators of myocardial stress and volume overload, whereas multi-marker strategies integrating ST2, Galectin-3, and Copeptin provide deeper insight into HF pathophysiology and potential therapeutic targets (215–219).

Intervention strategies have expanded beyond conventional therapy. SGLT2 inhibitors have demonstrated reductions in HF hospitalization and mortality, potentially through modulation of myocardial inflammation and the STAT1-STING-mediated cellular senescence pathway (220, 221). Lifestyle interventions, including adherence to a Mediterranean diet, can improve metabolic profiles and reduce systemic inflammation (222). Cutting-edge biological approaches, such as targeting activated CFs, gene- and cell-based therapies, and recombinant human ACE2 administration, aim to repair or mitigate pathological cardiac remodeling (223, 224). Integrating multi-omics, circulating biomarkers, and clinical variables facilitates precision management, particularly in patients with HFpEF, renal comorbidities, or metabolic disturbances (225–228).

Clinical studies indicate that rapid initiation and titration of guideline-directed therapies in acute HF reduces short-term mortality and rehospitalization, whereas strict sodium restriction in chronic HF has not shown significant outcome improvement (229, 230). Mechanism-targeted interventions, including IL-1/IL-6 blockade, corticosteroids, and colchicine, show potential in acute HF, while triacylglycerol supplementation demonstrates efficacy in triglyceride deposit cardiomyopathy (231, 232). Combining genetic markers, advanced imaging, and artificial intelligence-assisted diagnostics can optimize patient stratification and individualized therapeutic strategies (233). Collectively, emerging biological and personalized therapies provide multidimensional approaches to improve outcomes and slow disease progression in HF patients.

## 5 Future perspectives and conclusions

Although significant advances have been made in delineating the pathophysiological mechanisms and therapeutic strategies for HF, several challenges persist. Current treatments predominantly target neurohormonal pathways (e.g., RAAS and SNS), yet they only partially mitigate maladaptive cardiac remodeling and fail to adequately address the phenotypic heterogeneity observed in HFpEF patients (160, 234). Furthermore, limited mechanistic understanding of metabolic dysregulation, inflammation, and MVD constrains the development of fully effective interventions. Emerging strategies integrating mechanistic insights and advanced technologies may overcome these limitations. Machine learning–based models have demonstrated superior predictive accuracy for hospitalization and mortality by combining clinical parameters with biomarker and imaging data (235). Multi-omics approaches, including transcriptomic, proteomic, and metabolomic profiling, have revealed novel pathways involved in cardiomyocyte energy metabolism, iron handling, and ECM remodeling, offering potential targets for personalized therapy (74, 236).

Building on these developments, future research should prioritize integrating omics-derived biomarkers with machine learning and systems biology tools to refine risk prediction models and uncover mechanistically distinct HF subgroups. Such integrative frameworks may enable dynamic, individualized monitoring and more accurate identification of patients who are likely to respond to specific therapeutic modalities (236). In addition, developing personalized therapeutic approaches for distinct HF phenotypes—including HFpEF subgroups defined by metabolic dysfunction, systemic inflammation, or microvascular disease—will be crucial. Aligning therapeutic selection with molecular signatures and comorbidity profiles may help overcome the historical limitations of “one-size-fits-all” treatment paradigms (237). Finally, future clinical trial designs should better incorporate real-world patient complexity, including frailty, multimorbidity, and sex-specific differences that influence treatment response and tolerability. Adaptive and phenotype-stratified trial frameworks may enhance the evaluation of both established and emerging therapies, while digital health tools and patient-reported outcomes can improve longitudinal assessment of functional status and quality of life (238).

Together, these directions underscore the importance of integrating high-resolution mechanistic data with patient-specific phenotyping to enable precision-guided interventions. Translational studies combining experimental models, omics-based biomarker discovery, and clinical validation are expected to refine risk stratification, optimize therapeutic selection, and ultimately improve functional outcomes and survival in HF.

## Glossary

4EBP1: eukaryotic translation initiation factor 4E binding protein 1
ac4C: N4-acetylcytidine
ACE: angiotensin-converting enzyme
ACE2: angiotensin-converting enzyme 2
AF: atrial fibrillation
AKT: protein kinase B
AMPK: AMP-activated protein kinase
Ang1–7: angiotensin 1–7
ARBs: angiotensin II receptor blockers
ARNIs: angiotensin receptor–neprilysin inhibitors
AT1: angiotensin II type 1 receptor
AT1A: angiotensin II type 1A receptor
ATG5: autophagy related 5
AV: atrioventricular
BAG3: BCL2-associated athanogene 3
BET: bromodomain and extra-terminal proteins
Bmi1: B-cell-specific moloney murine leukemia virus integration site 1
BRD4: bromodomain-containing protein 4
CASA: chaperone-assisted selective autophagy
CCM: cardiac contractility modulation
CD47: cluster of differentiation 47
CFs: cardiac fibroblasts
circRNA: circular RNA
CMD: coronary microvascular dysfunction
CR: cardiac rehabilitation
CRT: cardiac resynchronization therapy
CXCL10: C-X-C motif chemokine ligand 10
DCM: dilated cardiomyopathy
ECM: extracellular matrix
Fe–S: iron–sulfur cluster
GDF-15: growth differentiation factor 15
GLP-1: glucagon-like peptide-1
GPX4: glutathione peroxidase 4
GSH: glutathione
GSHPx: glutathione peroxidase
HAND1: heart and neural crest derivatives expressed 1
HCTR: hybrid comprehensive telerehabilitation
HDAC: histone deacetylase
HF: heart failure
HFmrEF: heart failure with mildly reduced ejection fraction
HFpEF: heart failure with preserved ejection fraction
HFrEF: heart failure with reduced ejection fraction
HIIT: high-intensity interval training
HMGB1: high-mobility group box 1
HO-1: heme oxygenase-1
HSP27/HSP70: heat shock protein 27/70
ICD: implantable cardioverter-defibrillator
IGFBP7: insulin-like growth factor binding protein 7
IL-1: interleukin-1
IL-10: interleukin-10
IL-6: interleukin-6
Keap1: kelch-like ECH-associated protein 1
LBBB: left bundle branch block
LCZ696: sacubitril/valsartan
lncRNA: long non-coding RNA
LVAD: left ventricular assist device
LVEF: left ventricular ejection fraction
MAO: monoamine oxidases
MAPK: mitogen-activated protein kinase
Mas R: mas receptor
MCS: mechanical circulatory support
MEF2: myocyte enhancer factor 2
MFRN1: mitoferrin 1
MICT: moderate-intensity continuous training
miRNA: microRNA
MitoDAMPs: mitochondrial damage-associated molecular patterns
MLF1: myeloid leukemia factor 1
MR: mitral regurgitation
MRA: mineralocorticoid receptor antagonist
mTOR: mammalian target of rapamycin
MVD: microvascular dysfunction
myoFbs: myofibroblasts
NAD^+^: nicotinamide adenine dinucleotide
NAT10: N-acetyltransferase 10
NF-κB: nuclear factor-kappa B
NHANES: national health and nutrition examination survey
NLRP3: NOD-like receptor pyrin domain-containing protein 3
NO: nitric oxide
NOS: nitric oxide synthases
Nox2/Nox4: NADPH oxidase 2/4
NP: natriuretic peptide
NR: nicotinamide riboside
Nrf2: NF-E2-related factor 2
NYHA: New York heart association
PBMCs: peripheral blood mononuclear cells
PI3K: phosphatidylinositol 3-kinase
PKG: protein kinase G
PQC: protein quality control
RAAS: renin–angiotensin–aldosterone system
RNS: reactive nitrogen species
ROCK2: rho-associated protein kinase 2
ROR2: receptor tyrosine kinase-like orphan receptor 2
ROS: reactive oxygen species
SGLT2: sodium–glucose cotransporter 2
SGLT2i: sodium–glucose cotransporter 2 inhibitor
SII: systemic immune-inflammation index
SLC25A3: solute carrier family 25 member 3
Smad: Sma and mad related proteins
SNS: sympathetic nervous system
SOD: superoxide dismutase
ST2: suppression of tumorigenicity 2
STAT1: signal transducer and activator of transcription 1
STING: stimulator of interferon genes
TCA: tricarboxylic acid cycle
TGF-*β*: transforming growth factor-*β*
TLR4: toll-like receptor 4
TNFR1: tumor necrosis factor receptor 1
TNF-α: tumor necrosis factor-α
TRIM24: tripartite motif containing 24
Trx/TrxR: thioredoxin/thioredoxin reductase
Txlnb: taxilin beta
UPAR: urokinase-type plasminogen activator receptor
VO_2_peak: peak oxygen uptake
VV: interventricular
WHF: worsening heart failure
Wisper: wound and scar-associated lncRNA
XOR: xanthine oxidoreductase
Zip13: zinc transporter 13

## References

[1] Bozkurt B Coats A Tsutsui H. Universal definition and classification of heart failure. J Card Fail. (2021) 27(4):387–413. 10.1016/j.cardfail.2021.01.022 33663906

[2] Heidenreich PA Bozkurt B Aguilar D Allen LA Byun JJ Colvin MM 2022 AHA/ACC/HFSA guideline for the management of heart failure: a report of the American college of cardiology/American heart association joint committee on clinical practice guidelines. Circulation. (2022) 145:e895–e1032. 10.1161/CIR.0000000000001063 35363499

[3] Maryam Varghese TP Tazneem B. Unraveling the complex pathophysiology of heart failure: insights into the role of renin-angiotensin-aldosterone system (RAAS) and sympathetic nervous system (SNS). Curr Probl Cardiol. (2024) 49:102411. 10.1016/j.cpcardiol.2024.102411 38246316

[4] Abdin A Bohm M Shahim B Karlstrom P Kulenthiran S Skouri H Heart failure with preserved ejection fraction epidemiology, pathophysiology, diagnosis and treatment strategies. Int J Cardiol. (2024) 412:132304. 10.1016/j.ijcard.2024.132304 38944348

[5] Laborante R Restivo A Mele D Di Francesco M Ferreira JP Vasques-Novoa F Device-based strategies for monitoring congestion and guideline-directed therapy in heart failure: the who, when and how of personalised care. Card Fail Rev. (2025) 11:e11. 10.15420/cfr.2025.01 40458286 PMC12127967

[6] Castiglione V Gentile F Ghionzoli N Chiriaco M Panichella G Aimo A Pathophysiological rationale and clinical evidence for neurohormonal modulation in heart failure with preserved ejection fraction. Card Fail Rev. (2023) 9:e09. 10.15420/cfr.2022.23 37427009 PMC10326668

[7] Juillière Y Venner C Filippetti L Popovic B Huttin O Selton-Suty C. Heart failure with preserved ejection fraction: a systemic disease linked to multiple comorbidities, targeting new therapeutic options. Arch Cardiovasc Dis. (2018) 111:766–81. 10.1016/j.acvd.2018.04.007 29960837

[8] Palaparthi EC Padala T Singamaneni R Manaswini R Kantula A Aditya Reddy P Emerging therapeutic strategies for heart failure: a comprehensive review of novel pharmacological and molecular targets. Cureus. (2025) 17:e81573. 10.7759/cureus.81573 40313442 PMC12045464

[9] Koontalay A Botti M Hutchinson A. Narrative synthesis of the effectiveness and characteristics of heart failure disease self-management support programmes. ESC Heart Fail. (2024) 11:1329–40. 10.1002/ehf2.14701 38311880 PMC11098667

[10] Schwinger RHG. Pathophysiology of heart failure. Cardiovasc Diagn Ther. (2021) 11:263–76. 10.21037/cdt-20-302 33708498 PMC7944197

[11] Jia G Aroor AR Hill MA Sowers JR. Role of renin-angiotensin-aldosterone system activation in promoting cardiovascular fibrosis and stiffness. Hypertension. (2018) 72:537–48. 10.1161/HYPERTENSIONAHA.118.11065 29987104 PMC6202147

[12] Volpe M Tocci G Pagannone E. Activation of the renin-angiotensin-aldosterone system in heart failure. Ital Heart J. (2005) 6(1):16S–23. Available online at: https://pubmed.ncbi.nlm.nih.gov/15945296/15945296

[13] Harada K Sugaya T Murakami K Yazaki Y Komuro I. Angiotensin II type 1A receptor knockout mice display less left ventricular remodeling and improved survival after myocardial infarction. Circulation. (1999) 100:2093–9. 10.1161/01.cir.100.20.2093 10562266

[14] Wozniak M Tyrankiewicz U Drelicharz L Skorka T Jablonska M Heinze-Paluchowska S The effect of the renin-angiotensin-aldosterone system inhibition on myocardial function in early and late phases of dilated cardiomyopathy in Tgaq*44 mice. Kardiol Pol. (2013) 71:730–7. 10.5603/KP.2013.0161 23907907

[15] Chen H Yu B Guo X Hua H Cui F Guan Y Chronic intermittent hypobaric hypoxia decreases high blood pressure by stabilizing the vascular renin-angiotensin system in spontaneously hypertensive rats. Front Physiol. (2021) 12:639454. 10.3389/fphys.2021.639454 33841179 PMC8024534

[16] Hawlitschek C Brendel J Gabriel P Schierle K Salameh A Zimmer HG Antihypertensive and cardioprotective effects of different monotherapies and combination therapies in young spontaneously hypertensive rats—a pilot study. Saudi J Biol Sci. (2022) 29:339–45. 10.1016/j.sjbs.2021.08.093 35002427 PMC8716903

[17] Yi X Abas R Raja Muhammad Rooshdi RAW Yan J Liu C Yang C Time-restricted feeding reduced blood pressure and improved cardiac structure and function by regulating both circulating and local renin-angiotensin systems in spontaneously hypertensive rat model. PLoS One. (2025) 20:e0321078. 10.1371/journal.pone.0321078 40179126 PMC11967951

[18] McMurray JJ Packer M Desai AS Gong J Lefkowitz MP Rizkala AR Dual angiotensin receptor and neprilysin inhibition as an alternative to angiotensin-converting enzyme inhibition in patients with chronic systolic heart failure: rationale for and design of the prospective comparison of ARNI with ACEI to determine impact on global mortality and morbidity in heart failure trial (PARADIGM-HF). Eur J Heart Fail. (2013) 15:1062–73. 10.1093/eurjhf/hft052 23563576 PMC3746839

[19] Lind L Riserus U Elmstahl S Arnlov J Michaelsson K Titova OE. Combinations of BMI and metabolic syndrome and the risk of myocardial infarction, stroke, and heart failure. Nutr Metab Cardiovasc Dis. (2025) 35:104102. 10.1016/j.numecd.2025.104102 40414765

[20] Menghoum N Badii MC Leroy M Parra M Roy C Lejeune S Exploring the impact of metabolic comorbidities on epicardial adipose tissue in heart failure with preserved ejection fraction. Cardiovasc Diabetol. (2025) 24:134. 10.1186/s12933-025-02688-7 40121452 PMC11929347

[21] Jhund PS Talebi A Henderson AD Claggett BL Vaduganathan M Desai AS Mineralocorticoid receptor antagonists in heart failure: an individual patient level meta-analysis. Lancet. (2024) 404:1119–31. 10.1016/S0140-6736(24)01733-1 39232490

[22] Desai AS Vaduganathan M Claggett BL Kulac IJ Jhund PS Cunningham J Finerenone in patients with a recent worsening heart failure event: the FINEARTS-HF trial. J Am Coll Cardiol. (2025) 85:106–16. 10.1016/j.jacc.2024.09.004 39352340

[23] Butt JH Jhund PS Henderson AD Claggett BL Desai AS Viswanathan P Finerenone and new-onset diabetes in heart failure: a prespecified analysis of the FINEARTS-HF trial. Lancet Diabetes Endocrinol. (2025) 13:107–18. 10.1016/S2213-8587(24)00309-7 39818225

[24] Yeoh SE Osmanska J Petrie MC Brooksbank KJM Clark AL Docherty KF Dapagliflozin vs. metolazone in heart failure resistant to loop diuretics. Eur Heart J. (2023) 44:2966–77. 10.1093/eurheartj/ehad341 37210742 PMC10424881

[25] Hundertmark MJ Adler A Antoniades C Coleman R Griffin JL Holman RR Assessment of cardiac energy metabolism, function, and physiology in patients with heart failure taking empagliflozin: the randomized, controlled EMPA-VISION trial. Circulation. (2023) 147:1654–69. 10.1161/CIRCULATIONAHA.122.062021 37070436 PMC10212585

[26] Kitzman DW Lewis GD Pandey A Borlaug BA Sauer AJ Litwin SE A novel controlled metabolic accelerator for the treatment of obesity-related heart failure with preserved ejection fraction: rationale and design of the Phase 2a HuMAIN trial. Eur J Heart Fail. (2024) 26:2013–24. 10.1002/ejhf.3305 38924328 PMC11704968

[27] Mueller S Winzer EB Duvinage A Gevaert AB Edelmann F Haller B Effect of high-intensity interval training, moderate continuous training, or guideline-based physical activity advice on peak oxygen consumption in patients with heart failure with preserved ejection fraction: a randomized clinical trial. JAMA. (2021) 325:542–51. 10.1001/jama.2020.26812 33560320 PMC7873782

[28] Besnier F Labrunee M Richard L Faggianelli F Kerros H Soukarie L Short-term effects of a 3-week interval training program on heart rate variability in chronic heart failure. A randomised controlled trial. Ann Phys Rehabil Med. (2019) 62:321–8. 10.1016/j.rehab.2019.06.013 31352063

[29] Wang C Xing J Zhao B Wang Y Zhang L Wang Y The effects of high-intensity interval training on exercise capacity and prognosis in heart failure and coronary artery disease: a systematic review and meta-analysis. Cardiovasc Ther. (2022) 2022:4273809. 10.1155/2022/4273809 35801132 PMC9203221

[30] Wisloff U Stoylen A Loennechen JP Bruvold M Rognmo O Haram PM Superior cardiovascular effect of aerobic interval training versus moderate continuous training in heart failure patients: a randomized study. Circulation. (2007) 115:3086–94. 10.1161/CIRCULATIONAHA.106.675041 17548726

[31] Yamamoto S Okamura M Akashi YJ Tanaka S Shimizu M Tsuchikawa Y Impact of long-term exercise-based cardiac rehabilitation in patients with chronic heart failure- A systematic review and meta-analysis. Circ J. (2024) 88:1360–71. 10.1253/circj.CJ-23-0820 38220206

[32] Piotrowicz E Pencina MJ Opolski G Zareba W Banach M Kowalik I Effects of a 9-week hybrid comprehensive telerehabilitation program on long-term outcomes in patients with heart failure: the telerehabilitation in heart failure patients (TELEREH-HF) randomized clinical trial. JAMA Cardiol. (2020) 5:300–8. 10.1001/jamacardio.2019.5006 31734701 PMC6865325

[33] Gama F Rocha B Aguiar C Strong C Freitas P Brizido C Exercise oscillatory ventilation improves heart failure prognostic scores. Heart Lung Circ. (2023) 32:949–57. 10.1016/j.hlc.2023.04.291 37330375

[34] Lewis GD Malhotra R Hernandez AF McNulty SE Smith A Felker GM Effect of oral iron repletion on exercise capacity in patients with heart failure with reduced ejection fraction and iron deficiency: the IRONOUT HF randomized clinical trial. JAMA. (2017) 317:1958–66. 10.1001/jama.2017.5427 28510680 PMC5703044

[35] Zhou B Wang DD Qiu Y Airhart S Liu Y Stempien-Otero A Boosting NAD level suppresses inflammatory activation of PBMCs in heart failure. J Clin Invest. (2020) 130:6054–63. 10.1172/JCI138538 32790648 PMC7598081

[36] Li H Wang X Zhang Y Yang Y Zhang JZ Zhou B. SLC39A13 Regulates heart function via mitochondrial iron homeostasis maintenance. Circ Res. (2025) 137(6):e144–56. 10.1161/CIRCRESAHA.125.326201 40726388

[37] Zhang H Jamieson KL Grenier J Nikhanj A Tang Z Wang F Myocardial iron deficiency and mitochondrial dysfunction in advanced heart failure in humans. J Am Heart Assoc. (2022) 11:e022853. 10.1161/JAHA.121.022853 35656974 PMC9238720

[38] Hoes MF Grote Beverborg N Kijlstra JD Kuipers J Swinkels DW Giepmans BNG Iron deficiency impairs contractility of human cardiomyocytes through decreased mitochondrial function. Eur J Heart Fail. (2018) 20:910–9. 10.1002/ejhf.1154 29484788 PMC5993224

[39] Chiang S Braidy N Maleki S Lal S Richardson DR Huang ML. Mechanisms of impaired mitochondrial homeostasis and NAD(+) metabolism in a model of mitochondrial heart disease exhibiting redox active iron accumulation. Redox Biol. (2021) 46:102038. 10.1016/j.redox.2021.102038 34416478 PMC8379503

[40] Li S Zhang J Fu W Cao J Li Z Tian X Mitochondrial transplantation rescues Ca(2+) homeostasis imbalance and myocardial hypertrophy in SLC25A3-related hypertrophic cardiomyopathy. Cell Rep. (2024) 43:115065. 10.1016/j.celrep.2024.115065 39671292

[41] Wang YT Moura AK Zuo R Wang Z Roudbari K Hu JZ Defective lipid droplet biogenesis exacerbates oleic acid-induced cellular homeostasis disruption and ferroptosis in mouse cardiac endothelial cells. Cell Death Discov. (2025) 11:374. 10.1038/s41420-025-02669-5 40783384 PMC12335489

[42] Angermann CE Sehner S Gerhardt LMS Santos-Gallego CG Requena-Ibanez JA Zeller T Anaemia predicts iron homoeostasis dysregulation and modulates the response to empagliflozin in heart failure with reduced ejection fraction: the EMPATROPISM-FE trial. Eur Heart J. (2025) 46:1507–23. 10.1093/eurheartj/ehae917 39907687 PMC12011522

[43] Charles-Edwards G Amaral N Sleigh A Ayis S Catibog N McDonagh T Effect of iron isomaltoside on skeletal muscle energetics in patients with chronic heart failure and iron deficiency. Circulation. (2019) 139:2386–98. 10.1161/CIRCULATIONAHA.118.038516 30776909

[44] Packer M Ferreira JP Butler J Filippatos G Januzzi JL Jr. Gonzalez Maldonado S Reaffirmation of mechanistic proteomic signatures accompanying SGLT2 inhibition in patients with heart failure: a validation cohort of the EMPEROR program. J Am Coll Cardiol. (2024) 84:1979–94. 10.1016/j.jacc.2024.07.013 39217550

[45] van der Meer P van der Wal HH Melenovsky V. Mitochondrial function, skeletal muscle metabolism, and iron deficiency in heart failure. Circulation. (2019) 139:2399–402. 10.1161/CIRCULATIONAHA.119.040134 31107619

[46] He Z Gao B Deng Y Wu J Hu X Qin Z. Associations between systemic immune-inflammation index and heart failure: a cross-sectional study. Medicine. (2024) 103:e40096. 10.1097/MD.0000000000040096 39432602 PMC11495792

[47] Zheng H Yin Z Luo X Zhou Y Zhang F Guo Z. Associations between systemic immunity-inflammation index and heart failure: evidence from the NHANES 1999–2018. Int J Cardiol. (2024) 395:131400. 10.1016/j.ijcard.2023.131400 37769969

[48] Heidenreich P. Inflammation and heart failure: therapeutic or diagnostic opportunity? J Am Coll Cardiol. (2017) 69:1286–7. 10.1016/j.jacc.2017.01.013 28279295

[49] Sanders-van Wijk S Tromp J Beussink-Nelson L Hage C Svedlund S Saraste A Proteomic evaluation of the comorbidity-inflammation paradigm in heart failure with preserved ejection fraction: results from the PROMIS-HFpEF study. Circulation. (2020) 142:2029–44. 10.1161/CIRCULATIONAHA.120.045810 33034202 PMC7686082

[50] de Boer RA. Myeloperoxidase in heart failure with preserved ejection fraction: a target against inflammation? JACC Heart Fail. (2023) 11:788–90. 10.1016/j.jchf.2023.04.009 37178083

[51] Schiattarella GG Rodolico D Hill JA. Metabolic inflammation in heart failure with preserved ejection fraction. Cardiovasc Res. (2021) 117:423–34. 10.1093/cvr/cvaa217 32666082 PMC8599724

[52] Dong X Jiang J Lin Z Wen R Zou L Luo T Nuanxinkang protects against ischemia/reperfusion-induced heart failure through regulating IKKbeta/IkappaBalpha/NF-kappaB-mediated macrophage polarization. Phytomedicine. (2022) 101:154093. 10.1016/j.phymed.2022.154093 35447422

[53] Benedetti R Chianese U Papulino C Scisciola L Cortese M Formisano P Unlocking the power of empagliflozin: rescuing inflammation in hyperglycaemia-exposed human cardiomyocytes through comprehensive multi-level analysis. Eur J Heart Fail. (2025) 27:844–56. 10.1002/ejhf.3566 39809551 PMC12103962

[54] Wu Q Yao Q Hu T Yu J Jiang K Wan Y Dapagliflozin protects against chronic heart failure in mice by inhibiting macrophage-mediated inflammation, independent of SGLT2. Cell Rep Med. (2023) 4:101334. 10.1016/j.xcrm.2023.101334 38118414 PMC10772464

[55] Guo W Zhao L Huang W Chen J Zhong T Yan S Sodium-glucose cotransporter 2 inhibitors, inflammation, and heart failure: a two-sample Mendelian randomization study. Cardiovasc Diabetol. (2024) 23:118. 10.1186/s12933-024-02210-5 38566143 PMC10986088

[56] Kittipibul V Fudim M. Tackling inflammation in heart failure with preserved ejection fraction: resurrection of vagus nerve stimulation? J Am Heart Assoc. (2022) 11:e024481. 10.1161/JAHA.121.024481 35023352 PMC9238495

[57] Stavrakis S Elkholey K Morris L Niewiadomska M Asad ZUA Humphrey MB. Neuromodulation of inflammation to treat heart failure with preserved ejection fraction: a pilot randomized clinical trial. J Am Heart Assoc. (2022) 11:e023582. 10.1161/JAHA.121.023582 35023349 PMC9238491

[58] Bhattarai U Xu R He X Pan L Niu Z Wang D High selenium diet attenuates pressure overload-induced cardiopulmonary oxidative stress, inflammation, and heart failure. Redox Biol. (2024) 76:103325. 10.1016/j.redox.2024.103325 39197316 PMC11399737

[59] Xing Z Chen J Yu T Li X Dong W Peng C Aconitum carmichaelii Debx. Attenuates heart failure through inhibiting inflammation and abnormal vascular remodeling. Int J Mol Sci. (2023) 24:5838. 10.3390/ijms24065838 36982912 PMC10059042

[60] Aimo A Borrelli C Vergaro G Piepoli MF Caterina AR Mirizzi G Targeting mitochondrial dysfunction in chronic heart failure: current evidence and potential approaches. Curr Pharm Des. (2016) 22:4807–22. 10.2174/1381612822666160701075027 27396600

[61] Aimo A Castiglione V Borrelli C Saccaro LF Franzini M Masi S Oxidative stress and inflammation in the evolution of heart failure: from pathophysiology to therapeutic strategies. Eur J Prev Cardiol. (2020) 27:494–510. 10.1177/2047487319870344 31412712

[62] D'Oria R Schipani R Leonardini A Natalicchio A Perrini S Cignarelli A The role of oxidative stress in cardiac disease: from physiological response to injury factor. Oxid Med Cell Longev. (2020) 2020:5732956. 10.1155/2020/5732956 32509147 PMC7244977

[63] Li T Wang N Yi D Xiao Y Li X Shao B ROS-mediated ferroptosis and pyroptosis in cardiomyocytes: an update. Life Sci. (2025) 370:123565. 10.1016/j.lfs.2025.123565 40113077

[64] Tsutsui H Kinugawa S Matsushima S. Oxidative stress and heart failure. Am J Physiol Heart Circ Physiol. (2011) 301:H2181–2190. 10.1152/ajpheart.00554.2011 21949114

[65] Fu J Su C Ge Y Ao Z Xia L Chen Y PDE4D Inhibition ameliorates cardiac hypertrophy and heart failure by activating mitophagy. Redox Biol. (2025) 81:103563. 10.1016/j.redox.2025.103563 40015131 PMC11909752

[66] Ren H Hu W Jiang T Yao Q Qi Y Huang K. Mechanical stress induced mitochondrial dysfunction in cardiovascular diseases: novel mechanisms and therapeutic targets. Biomed Pharmacother. (2024) 174:116545. 10.1016/j.biopha.2024.116545 38603884

[67] Wang J Li S Yu H Gao D. Oxidative stress regulates cardiomyocyte energy metabolism through the IGF2BP2-dynamin2 signaling pathway. Biochem Biophys Res Commun. (2022) 624:134–40. 10.1016/j.bbrc.2022.07.089 35940126

[68] Ye X Lin ZJ Hong GH Wang ZM Dou RT Lin JY Pyroptosis inhibitors MCC950 and VX-765 mitigate myocardial injury by alleviating oxidative stress, inflammation, and apoptosis in acute myocardial hypoxia. Exp Cell Res. (2024) 438:114061. 10.1016/j.yexcr.2024.114061 38692345

[69] Hou X Hu G Wang H Yang Y Sun Q Bai X. Acot1 overexpression alleviates heart failure by inhibiting oxidative stress and cardiomyocyte apoptosis through the Keap1-Nrf2 pathway. Exp Anim. (2025). 10.1538/expanim.24-0129 PMC12861710 40240160

[70] Wang Z Zhang Y Wang L Yang C Yang H. NBP relieves cardiac injury and reduce oxidative stress and cell apoptosis in heart failure mice by activating Nrf2/HO-1/Ca(2+)-SERCA2a axis. Evid Based Complement Alternat Med. (2022) 2022:7464893. 10.1155/2022/7464893 36452141 PMC9705093

[71] Zhu K Fan R Cao Y Yang W Zhang Z Zhou Q Glycyrrhizin attenuates myocardial ischemia reperfusion injury by suppressing inflammation, oxidative stress, and ferroptosis via the HMGB1-TLR4-GPX4 pathway. Exp Cell Res. (2024) 435:113912. 10.1016/j.yexcr.2024.113912 38176464

[72] Peoples JN Saraf A Ghazal N Pham TT Kwong JQ. Mitochondrial dysfunction and oxidative stress in heart disease. Exp Mol Med. (2019) 51:1–13. 10.1038/s12276-019-0355-7 31857574 PMC6923355

[73] Czubryt MP Hale TM. Cardiac fibrosis: pathobiology and therapeutic targets. Cell Signal. (2021) 85:110066. 10.1016/j.cellsig.2021.110066 34146658 PMC8355135

[74] Ghazal R Wang M Liu D Tschumperlin DJ Pereira NL. Cardiac fibrosis in the multi-omics era: implications for heart failure. Circ Res. (2025) 136:773–802. 10.1161/CIRCRESAHA.124.325402 40146800 PMC11949229

[75] Liu M Lopez de Juan Abad B Cheng K. Cardiac fibrosis: myofibroblast-mediated pathological regulation and drug delivery strategies. Adv Drug Deliv Rev. (2021) 173:504–19. 10.1016/j.addr.2021.03.021 33831476 PMC8299409

[76] Andersen S Nielsen-Kudsk JE Vonk Noordegraaf A de Man FS. Right ventricular fibrosis. Circulation. (2019) 139:269–85. 10.1161/CIRCULATIONAHA.118.035326 30615500

[77] Schimmel K Ichimura K Reddy S Haddad F Spiekerkoetter E. Cardiac fibrosis in the pressure overloaded left and right ventricle as a therapeutic target. Front Cardiovasc Med. (2022) 9:886553. 10.3389/fcvm.2022.886553 35600469 PMC9120363

[78] Li R Frangogiannis NG. Integrins in cardiac fibrosis. J Mol Cell Cardiol. (2022) 172:1–13. 10.1016/j.yjmcc.2022.07.006 35872324

[79] Yao Y Hu C Song Q Li Y Da X Yu Y ADAMTS16 Activates latent TGF-beta, accentuating fibrosis and dysfunction of the pressure-overloaded heart. Cardiovasc Res. (2020) 116:956–69. 10.1093/cvr/cvz187 31297506 PMC7868664

[80] Andenaes K Lunde IG Mohammadzadeh N Dahl CP Aronsen JM Strand ME The extracellular matrix proteoglycan fibromodulin is upregulated in clinical and experimental heart failure and affects cardiac remodeling. PLoS One. (2018) 13:e0201422. 10.1371/journal.pone.0201422 30052659 PMC6063439

[81] Deng B Zhang Y Zhu C Wang Y Weatherford E Xu B Divergent actions of myofibroblast and myocyte beta(2)-Adrenoceptor in heart failure and fibrotic remodeling. Circ Res. (2023) 132:106–8. 10.1161/CIRCRESAHA.122.321816 36458552 PMC9985902

[82] Frangogiannis NG. Cardiac fibrosis: cell biological mechanisms, molecular pathways and therapeutic opportunities. Mol Aspects Med. (2019) 65:70–99. 10.1016/j.mam.2018.07.001 30056242

[83] Micheletti R Plaisance I Abraham BJ Sarre A Ting CC Alexanian M The long noncoding RNA Wisper controls cardiac fibrosis and remodeling. Sci Transl Med. (2017) 9:eaai9118. 10.1126/scitranslmed.aai9118 28637928 PMC5643582

[84] Li L Zhao Q Kong W. Extracellular matrix remodeling and cardiac fibrosis. Matrix Biol. (2018) 68–69:490–506. 10.1016/j.matbio.2018.01.013 29371055

[85] Segura AM Frazier OH Buja LM. Fibrosis and heart failure. Heart Fail Rev. (2014) 19:173–85. 10.1007/s10741-012-9365-4 23124941

[86] Farris SD Don C Helterline D Costa C Plummer T Steffes S Cell-specific pathways supporting persistent fibrosis in heart failure. J Am Coll Cardiol. (2017) 70:344–54. 10.1016/j.jacc.2017.05.040 28705316

[87] Pandey AK Bhatt DL Pandey A Marx N Cosentino F Pandey A Mechanisms of benefits of sodium-glucose cotransporter 2 inhibitors in heart failure with preserved ejection fraction. Eur Heart J. (2023) 44:3640–51. 10.1093/eurheartj/ehad389 37674356

[88] Travers JG Wennersten SA Pena B Bagchi RA Smith HE Hirsch RA HDAC inhibition reverses preexisting diastolic dysfunction and blocks covert extracellular matrix remodeling. Circulation. (2021) 143:1874–90. 10.1161/CIRCULATIONAHA.120.046462 33682427 PMC8884170

[89] Frangogiannis NG. Cardiac fibrosis. Cardiovasc Res. (2021) 117:1450–88. 10.1093/cvr/cvaa324 33135058 PMC8152700

[90] Travers JG Kamal FA Robbins J Yutzey KE Blaxall BC. Cardiac fibrosis: the fibroblast awakens. Circ Res. (2016) 118:1021–40. 10.1161/CIRCRESAHA.115.306565 26987915 PMC4800485

[91] Valiente-Alandi I Schafer AE Blaxall BC. Extracellular matrix-mediated cellular communication in the heart. J Mol Cell Cardiol. (2016) 91:228–37. 10.1016/j.yjmcc.2016.01.011 26778458 PMC4767504

[92] Bielawska M Warszynska M Stefanska M Blyszczuk P. Autophagy in heart failure: insights into mechanisms and therapeutic implications. J Cardiovasc Dev Dis. (2023) 10:352. 10.3390/jcdd10080352 37623365 PMC10456056

[93] Li J Zhang D Wiersma M Brundel B. Role of autophagy in proteostasis: friend and foe in cardiac diseases. Cells. (2018) 7:279. 10.3390/cells7120279 30572675 PMC6316637

[94] Nah J Zablocki D Sadoshima J. The role of autophagic cell death in cardiac disease. J Mol Cell Cardiol. (2022) 173:16–24. 10.1016/j.yjmcc.2022.08.362 36084743

[95] Tang Y Xu W Liu Y Zhou J Cui K Chen Y. Autophagy protects mitochondrial health in heart failure. Heart Fail Rev. (2024) 29:113–23. 10.1007/s10741-023-10354-x 37823952

[96] Oeing CU Mishra S Dunkerly-Eyring BL Ranek MJ. Targeting protein kinase G to treat cardiac proteotoxicity. Front Physiol. (2020) 11:858. 10.3389/fphys.2020.00858 32848832 PMC7399205

[97] Maroli G Schanzer A Gunther S Garcia-Gonzalez C Rupp S Schlierbach H Inhibition of autophagy prevents cardiac dysfunction at early stages of cardiomyopathy in Bag3-deficient hearts. J Mol Cell Cardiol. (2024) 193:53–66. 10.1016/j.yjmcc.2024.06.001 38838815

[98] Delalat S Sultana I Osman H Sieme M Zhazykbayeva S Herwig M Dysregulated inflammation, oxidative stress, and protein quality control in diabetic HFpEF: unraveling mechanisms and therapeutic targets. Cardiovasc Diabetol. (2025) 24:211. 10.1186/s12933-025-02734-4 40369521 PMC12080046

[99] Cui T Lai Y Janicki JS Wang X. Nuclear factor erythroid-2 related factor 2 (Nrf2)-mediated protein quality control in cardiomyocytes. Front Biosci. (2016) 21:192–202. 10.2741/4384 PMC4723105 26709769

[100] Kobak KA Zarzycka W King CJ Borowik AK Peelor FF 3rd Baehr LM Proteostatic imbalance drives the pathogenesis and age-related exacerbation of heart failure with preserved ejection fraction. JACC Basic Transl Sci. (2025) 10:475–97. 10.1016/j.jacbts.2024.11.006 40306856 PMC12134603

[101] Zarzycka W Kobak KA King CJ Peelor FF Miller BF Chiao YA. Hyperactive mTORC1/4EBP1 signaling dysregulates proteostasis and accelerates cardiac aging. *bioRxiv*. (2024). 10.1101/2024.05.13.594044 PMC11979070 39379739

[102] Hartman H Uy G Uchida K Scarborough EA Yang Y Barr E ROR2 drives right ventricular heart failure via disruption of proteostasis. *bioRxiv*. (2025). 10.1101/2025.02.01.635961

[103] McLendon JM Zhang X Stein CS Baehr LM Bodine SC Boudreau RL. A specialized centrosome-proteasome axis mediates proteostasis and influences cardiac stress through Txlnb. *bioRxiv*. (2024). 10.1101/2024.02.12.580020 PMC12465015 40010430

[104] McLendon JM Zhang X Stein CS Baehr LM Bodine SC Boudreau RL. Gain and loss of the centrosomal protein taxilin-beta influences cardiac proteostasis and stress. J Mol Cell Cardiol. (2025) 201:56–69. 10.1016/j.yjmcc.2025.02.008 40010430 PMC12465015

[105] Neu M Deshpande A Borlepawar A Hammer E Alameldeen A Vocking P TRIM24 Regulates chromatin remodeling and calcium dynamics in cardiomyocytes. Cell Commun Signal. (2025) 23:312. 10.1186/s12964-025-02323-8 40598158 PMC12211185

[106] Fanjul V Jorge I Camafeita E Macias A Gonzalez-Gomez C Barettino A Identification of common cardiometabolic alterations and deregulated pathways in mouse and pig models of aging. Aging Cell. (2020) 19:e13203. 10.1111/acel.13203 32729659 PMC7511870

[107] Narayanan G Halim A Hu A Avin KG Lu T Zehnder D Molecular phenotyping and mechanisms of myocardial fibrosis in advanced chronic kidney disease. Kidney360. (2023) 4:1562–79. 10.34067/KID.0000000000000276 37858297 PMC10695648

[108] Olkowski AA Wojnarowicz C Laarveld B. Pathophysiology and pathological remodelling associated with dilated cardiomyopathy in broiler chickens predisposed to heart pump failure. Avian Pathol. (2020) 49:428–39. 10.1080/03079457.2020.1757620 32301624

[109] Li A Gao M Liu B Qin Y Chen L Liu H Mitochondrial autophagy: molecular mechanisms and implications for cardiovascular disease. Cell Death Dis. (2022) 13:444. 10.1038/s41419-022-04906-6 35534453 PMC9085840

[110] Cetrullo S D'Adamo S Tantini B Borzi RM Flamigni F. mTOR, AMPK, and Sirt1: key players in metabolic stress management. Crit Rev Eukaryot Gene Expr. (2015) 25:59–75. 10.1615/critreveukaryotgeneexpr.2015012975 25955819

[111] Maejima Y Titus AS Zablocki D Sadoshima J. Recent progress regarding the role of autophagy in cardiac disease. Cardiovasc Res. (2025):cvaf203. [Online ahead of print]. 10.1093/cvr/cvaf203 41144634

[112] Liao M Xie Q Zhao Y Yang C Lin C Wang G Main active components of Si-Miao-Yong-An decoction (SMYAD) attenuate autophagy and apoptosis via the PDE5A-AKT and TLR4-NOX4 pathways in isoproterenol (ISO)-induced heart failure models. Pharmacol Res. (2022) 176:106077. 10.1016/j.phrs.2022.106077 35026404

[113] Wang D Lv L Xu Y Jiang K Chen F Qian J Cardioprotection of Panax Notoginseng saponins against acute myocardial infarction and heart failure through inducing autophagy. Biomed Pharmacother. (2021) 136:111287. 10.1016/j.biopha.2021.111287 33485065

[114] Zhang X Wang Q Wang X Chen X Shao M Zhang Q Tanshinone IIA protects against heart failure post-myocardial infarction via AMPKs/mTOR-dependent autophagy pathway. Biomed Pharmacother. (2019) 112:108599. 10.1016/j.biopha.2019.108599 30798134

[115] Gao G Chen W Yan M Liu J Luo H Wang C Rapamycin regulates the balance between cardiomyocyte apoptosis and autophagy in chronic heart failure by inhibiting mTOR signaling. Int J Mol Med. (2020) 45:195–209. 10.3892/ijmm.2019.4407 31746373 PMC6889932

[116] Corsetti G Pasini E Romano C Chen-Scarabelli C Scarabelli TM Flati V How can malnutrition affect autophagy in chronic heart failure? Focus and perspectives. Int J Mol Sci. (2021) 22:3332. 10.3390/ijms22073332 33805128 PMC8036550

[117] Hu L Gao D Lv H Lian L Wang M Wang Y Finding new targets for the treatment of heart failure: endoplasmic reticulum stress and autophagy. J Cardiovasc Transl Res. (2023) 16:1349–56. 10.1007/s12265-023-10410-9 37432587

[118] Rush CJ Berry C Oldroyd KG Rocchiccioli JP Lindsay MM Touyz RM Prevalence of coronary artery disease and coronary microvascular dysfunction in patients with heart failure with preserved ejection fraction. JAMA Cardiol. (2021) 6:1130–43. 10.1001/jamacardio.2021.1825 34160566 PMC8223134

[119] Singh A Ashraf S Irfan H Venjhraj F Verma A Shaukat A Heart failure and microvascular dysfunction: an in-depth review of mechanisms, diagnostic strategies, and innovative therapies. Ann Med Surg. (2025) 87:616–26. 10.1097/MS9.0000000000002971 PMC11918592 40110322

[120] Ahmad A Corban MT Toya T Verbrugge FH Sara JD Lerman LO Coronary microvascular dysfunction is associated with exertional haemodynamic abnormalities in patients with heart failure with preserved ejection fraction. Eur J Heart Fail. (2021) 23:765–72. 10.1002/ejhf.2010 32949186

[121] Yang JH Obokata M Reddy YNV Redfield MM Lerman A Borlaug BA. Endothelium-dependent and independent coronary microvascular dysfunction in patients with heart failure with preserved ejection fraction. Eur J Heart Fail. (2020) 22:432–41. 10.1002/ejhf.1671 31840366

[122] Gamrat A Surdacki MA Chyrchel B Surdacki A. Endothelial dysfunction: a contributor to adverse cardiovascular remodeling and heart failure development in type 2 diabetes beyond accelerated atherogenesis. J Clin Med. (2020) 9:2090. 10.3390/jcm9072090 32635218 PMC7408687

[123] Cauwenberghs N Godderis S Sabovcik F Cornelissen V Kuznetsova T. Subclinical heart remodeling and dysfunction in relation to peripheral endothelial dysfunction: a general population study. Microcirculation. (2021) 28:e12731. 10.1111/micc.12731 34569675

[124] Shantsila E Wrigley BJ Blann AD Gill PS Lip GY. A contemporary view on endothelial function in heart failure. Eur J Heart Fail. (2012) 14:873–81. 10.1093/eurjhf/hfs066 22677484

[125] Balmain S Padmanabhan N Ferrell WR Morton JJ McMurray JJ. Differences in arterial compliance, microvascular function and venous capacitance between patients with heart failure and either preserved or reduced left ventricular systolic function. Eur J Heart Fail. (2007) 9:865–71. 10.1016/j.ejheart.2007.06.003 17644472

[126] Aguado-Alvaro LP Garitano N Pelacho B. Fibroblast diversity and epigenetic regulation in cardiac fibrosis. Int J Mol Sci. (2024) 25:6004. 10.3390/ijms25116004 38892192 PMC11172550

[127] Shi J Yang C Zhang J Zhao K Li P Kong C NAT10 is involved in cardiac remodeling through ac4C-mediated transcriptomic regulation. Circ Res. (2023) 133:989–1002. 10.1161/CIRCRESAHA.122.322244 37955115

[128] Gomes CPC Schroen B Kuster GM Robinson EL Ford K Squire IB Regulatory RNAs in heart failure. Circulation. (2020) 141:313–28. 10.1161/CIRCULATIONAHA.119.042474 31986093 PMC7012349

[129] Yang YL Li XW Chen HB Tang QD Li YH Xu JY Single-cell transcriptomics reveals writers of RNA modification-mediated immune microenvironment and cardiac resident Macro-MYL2 macrophages in heart failure. BMC Cardiovasc Disord. (2024) 24:432. 10.1186/s12872-024-04080-x 39152369 PMC11328403

[130] Ijaz T Burke MA. BET protein-mediated transcriptional regulation in heart failure. Int J Mol Sci. (2021) 22:6059. 10.3390/ijms22116059 34199719 PMC8199980

[131] Kim SY Zhang X Schiattarella GG Altamirano F Ramos TAR French KM Epigenetic reader BRD4 (bromodomain-containing protein 4) governs nucleus-encoded mitochondrial transcriptome to regulate cardiac function. Circulation. (2020) 142:2356–70. 10.1161/CIRCULATIONAHA.120.047239 33113340 PMC7736324

[132] Stratton MS Bagchi RA Felisbino MB Hirsch RA Smith HE Riching AS Dynamic chromatin targeting of BRD4 stimulates cardiac fibroblast activation. Circ Res. (2019) 125:662–77. 10.1161/CIRCRESAHA.119.315125 31409188 PMC7310347

[133] Feng Y Cai L Hong W Zhang C Tan N Wang M Rewiring of 3D chromatin topology orchestrates transcriptional reprogramming and the development of human dilated cardiomyopathy. Circulation. (2022) 145:1663–83. 10.1161/CIRCULATIONAHA.121.055781 35400201 PMC9251830

[134] Gi WT Haas J Sedaghat-Hamedani F Kayvanpour E Tappu R Lehmann DH Epigenetic regulation of alternative mRNA splicing in dilated cardiomyopathy. J Clin Med. (2020) 9:1499. 10.3390/jcm9051499 32429430 PMC7291244

[135] Gonzalez-Valdes I Hidalgo I Bujarrabal A Lara-Pezzi E Padron-Barthe L Garcia-Pavia P Bmi1 limits dilated cardiomyopathy and heart failure by inhibiting cardiac senescence. Nat Commun. (2015) 6:6473. 10.1038/ncomms7473 25751743 PMC5603726

[136] Lv J Chen Q Wang J Guo N Fang Y Guo Q Downregulation of MLF1 safeguards cardiomyocytes against senescence-associated chromatin opening. Nucleic Acids Res. (2025) 53:gkae1176. 10.1093/nar/gkae1176 39657728 PMC11754730

[137] Marques FZ Chu PY Ziemann M Kaspi A Kiriazis H Du XJ Age-related differential structural and transcriptomic responses in the hypertensive heart. Front Physiol. (2018) 9:817. 10.3389/fphys.2018.00817 30038575 PMC6046461

[138] Szulik MW Davis K Bakhtina A Azarcon P Bia R Horiuchi E Transcriptional regulation by methyltransferases and their role in the heart: highlighting novel emerging functionality. Am J Physiol Heart Circ Physiol. (2020) 319:H847–65. 10.1152/ajpheart.00382.2020 32822544 PMC7654657

[139] Cheng MD Li CL Pei XY Zhang YF Jia DD Zuo YB Integrative analysis of DNA methylome and transcriptome reveals epigenetic regulation of bisphenols-induced cardiomyocyte hypertrophy. Ecotoxicol Environ Saf. (2023) 263:115391. 10.1016/j.ecoenv.2023.115391 37611474

[140] Glezeva N Moran B Collier P Moravec CS Phelan D Donnellan E Targeted DNA methylation profiling of human cardiac tissue reveals novel epigenetic traits and gene deregulation across different heart failure patient subtypes. Circ Heart Fail. (2019) 12:e005765. 10.1161/CIRCHEARTFAILURE.118.005765 30798618

[141] Meder B Haas J Sedaghat-Hamedani F Kayvanpour E Frese K Lai A Epigenome-wide association study identifies cardiac gene patterning and a novel class of biomarkers for heart failure. Circulation. (2017) 136:1528–44. 10.1161/CIRCULATIONAHA.117.027355 28838933

[142] Jackson AO Rahman GA Yin K Long S. Enhancing matured stem-cardiac cell generation and transplantation: a novel strategy for heart failure therapy. J Cardiovasc Transl Res. (2021) 14:556–72. 10.1007/s12265-020-10085-6 33258081

[143] Junxi L Mocun Y Boda Z. Epigenetic and epitranscriptomic regulation of cardiac metabolism in aging and disease. J Cardiovasc Aging. (2025) 5:15. 10.20517/jca.2025.06

[144] Brand CS Lighthouse JK Trembley MA. Protective transcriptional mechanisms in cardiomyocytes and cardiac fibroblasts. J Mol Cell Cardiol. (2019) 132:1–12. 10.1016/j.yjmcc.2019.04.023 31042488 PMC6571165

[145] Shao X Zhang X Yang L Zhang R Zhu R Feng R. Integrated analysis of mRNA and microRNA expression profiles reveals differential transcriptome signature in ischaemic and dilated cardiomyopathy induced heart failure. Epigenetics. (2021) 16:917–32. 10.1080/15592294.2020.1827721 33016206 PMC8331008

[146] Piran S Liu P Morales A Hershberger RE. Where genome meets phenome: rationale for integrating genetic and protein biomarkers in the diagnosis and management of dilated cardiomyopathy and heart failure. J Am Coll Cardiol. (2012) 60:283–9. 10.1016/j.jacc.2012.05.005 22813604

[147] Maharaj R. Diastolic dysfunction and heart failure with a preserved ejection fraction: relevance in critical illness and anaesthesia. J Saudi Heart Assoc. (2012) 24:99–121. 10.1016/j.jsha.2012.01.004 23960679 PMC3727489

[148] Manolis AA Manolis TA Manolis AS. Neurohumoral activation in heart failure. Int J Mol Sci. (2023) 24:15472. 10.3390/ijms242015472 37895150 PMC10607846

[149] Checa-Ros A Okojie OJ D'Marco L. SGLT2 inhibitors: multifaceted therapeutic agents in cardiometabolic and renal diseases. Metabolites. (2025) 15:536. 10.3390/metabo15080536 40863154 PMC12388714

[150] Xu X Pang Y Fan X. Mitochondria in oxidative stress, inflammation and aging: from mechanisms to therapeutic advances. Signal Transduct Target Ther. (2025) 10:190. 10.1038/s41392-025-02253-4 40500258 PMC12159213

[151] Rolski F Maczewski M. Cardiac fibrosis: mechanistic discoveries linked to SGLT2 inhibitors. Pharmaceuticals. (2025) 18:313. 10.3390/ph18030313 40143092 PMC11944955

[152] Ma C Liu Y Fu Z. Implications of endoplasmic reticulum stress and autophagy in aging and cardiovascular diseases. Front Pharmacol. (2024) 15:1413853. 10.3389/fphar.2024.1413853 39119608 PMC11306071

[153] Numata G Takimoto E. Cyclic GMP and PKG signaling in heart failure. Front Pharmacol. (2022) 13:792798. 10.3389/fphar.2022.792798 35479330 PMC9036358

[154] Di Salvo TG Haldar SM. Epigenetic mechanisms in heart failure pathogenesis. Circ Heart Fail. (2014) 7:850–63. 10.1161/CIRCHEARTFAILURE.114.001193 25228320 PMC4169025

[155] Aldujeli A Tsai TY Haq A Tatarunas V Knokneris A Briedis K Impact of coronary microvascular dysfunction on functional left ventricular remodeling and diastolic dysfunction. J Am Heart Assoc. (2024) 13:e033596. 10.1161/JAHA.123.033596 38686863 PMC11179865

[156] Ansari RA Senapati SG Ahluwalia V Panjwani GAR Kaur A Yerrapragada G Artificial intelligence-guided neuromodulation in heart failure with preserved and reduced ejection fraction: mechanisms, evidence, and future directions. J Cardiovasc Dev Dis. (2025) 12:314. 10.3390/jcdd12080314 40863380 PMC12386544

[157] Mann DL Felker GM. Mechanisms and models in heart failure: a translational approach. Circ Res. (2021) 128:1435–50. 10.1161/CIRCRESAHA.121.318158 33983832 PMC8130816

[158] Chow SL Maisel AS Anand I Bozkurt B de Boer RA Felker GM Role of biomarkers for the prevention, assessment, and management of heart failure: a scientific statement from the American heart association. Circulation. (2017) 135:e1054–91. 10.1161/CIR.0000000000000490 28446515

[159] Fu S Ping P Wang F Luo L. Synthesis, secretion, function, metabolism and application of natriuretic peptides in heart failure. J Biol Eng. (2018) 12:2. 10.1186/s13036-017-0093-0 29344085 PMC5766980

[160] Hartupee J Mann DL. Neurohormonal activation in heart failure with reduced ejection fraction. Nat Rev Cardiol. (2017) 14:30–8. 10.1038/nrcardio.2016.163 27708278 PMC5286912

[161] Ameri P Bertero E Meliota G Cheli M Canepa M Brunelli C Neurohormonal activation and pharmacological inhibition in pulmonary arterial hypertension and related right ventricular failure. Heart Fail Rev. (2016) 21:539–47. 10.1007/s10741-016-9566-3 27206576

[162] Onuigbo MA. RAAS Inhibition and cardiorenal syndrome. Curr Hypertens Rev. (2014) 10:107–11. 10.2174/1573402111666141231144228 25549841

[163] Cruickshank JM. Beta-blockers and heart failure. Indian Heart J. (2010) 62:101–10. Available online at: https://pubmed.ncbi.nlm.nih.gov/21180298/21180298

[164] Metra M Cas LD di Lenarda A Poole-Wilson P. Beta-blockers in heart failure: are pharmacological differences clinically important? Heart Fail Rev. (2004) 9:123–30. 10.1023/B:HREV.0000046367.99002.a4 15516860

[165] Tobin SW Hashemi S Dadson K Turdi S Ebrahimian K Zhao J Heart failure and MEF2 transcriptome dynamics in response to beta-blockers. Sci Rep. (2017) 7:4476. 10.1038/s41598-017-04762-x 28667250 PMC5493616

[166] Fu S Chang Z Luo L Deng J. Therapeutic progress and knowledge basis on the natriuretic peptide system in heart failure. Curr Top Med Chem. (2019) 19:1850–66. 10.2174/1568026619666190826163536 31448711

[167] Khder Y Shi V McMurray JJV Lefkowitz MP. Sacubitril/valsartan (LCZ696) in heart failure. Handb Exp Pharmacol. (2017) 243:133–65. 10.1007/164_2016_77 28004291

[168] Nielsen PM Grimm D Wehland M Simonsen U Kruger M. The combination of valsartan and sacubitril in the treatment of hypertension and heart failure—an update. Basic Clin Pharmacol Toxicol. (2018) 122:9–18. 10.1111/bcpt.12912 28944989

[169] Volterrani M Iellamo F Senni M Piepoli MF. Therapeutic options of angiotensin receptor neprilysin inhibitors (ARNis) in chronic heart failure with reduced ejection fraction: beyond RAAS and sympathetic nervous system inhibition. Int J Cardiol. (2017) 226:132–5. 10.1016/j.ijcard.2016.04.180 27184730

[170] von Lueder TG Atar D Krum H. Current role of neprilysin inhibitors in hypertension and heart failure. Pharmacol Ther. (2014) 144:41–9. 10.1016/j.pharmthera.2014.05.002 24836726

[171] Rossignol P Zannad F Pitt B. Writing group of 10th global cardio vascular clinical trialist forum held on December 6th-7th in Paris F. Time to retrieve the best benefits from renin angiotensin aldosterone system (RAAS) inhibition in heart failure patients with reduced ejection fraction: lessons from randomized controlled trials and registries. Int J Cardiol. (2014) 177:731–3. 10.1016/j.ijcard.2014.11.004 25465821

[172] Vaduganathan M Fonarow GC Greene SJ Devore AD Albert NM Duffy CI Treatment persistence of renin-angiotensin-aldosterone-system inhibitors over time in heart failure with reduced ejection fraction. J Card Fail. (2022) 28:191–201. 10.1016/j.cardfail.2021.08.008 34428591

[173] Mascolo A di Mauro G Cappetta D De Angelis A Torella D Urbanek K Current and future therapeutic perspective in chronic heart failure. Pharmacol Res. (2022) 175:106035. 10.1016/j.phrs.2021.106035 34915125

[174] Nochioka K Sakata Y Shimokawa H. Combination therapy of renin angiotensin system inhibitors and beta-blockers in patients with heart failure. Adv Exp Med Biol. (2018) 1067:17–30. 10.1007/5584_2018_179 29542073

[175] Yamamoto K. beta-Blocker therapy in heart failure with preserved ejection fraction: importance of dose and duration. J Cardiol. (2015) 66:189–94. 10.1016/j.jjcc.2015.02.004 25881728

[176] Billing AM Kim YC Gullaksen S Schrage B Raabe J Hutzfeldt A Metabolic communication by SGLT2 inhibition. Circulation. (2024) 149:860–84. 10.1161/CIRCULATIONAHA.123.065517 38152989 PMC10922673

[177] Chen S Coronel R Hollmann MW Weber NC Zuurbier CJ. Direct cardiac effects of SGLT2 inhibitors. Cardiovasc Diabetol. (2022) 21:45. 10.1186/s12933-022-01480-1 35303888 PMC8933888

[178] Scheen AJ Bonnet F. Efficacy and safety profile of SGLT2 inhibitors in the elderly: how is the benefit/risk balance? Diabetes Metab. (2023) 49:101419. 10.1016/j.diabet.2023.101419 36640828

[179] Withaar C Meems LMG Markousis-Mavrogenis G Boogerd CJ Sillje HHW Schouten EM The effects of liraglutide and dapagliflozin on cardiac function and structure in a multi-hit mouse model of heart failure with preserved ejection fraction. Cardiovasc Res. (2021) 117:2108–24. 10.1093/cvr/cvaa256 32871009 PMC8318109

[180] Rizzo MR Di Meo I Polito R Auriemma MC Gambardella A di Mauro G Cognitive impairment and type 2 diabetes mellitus: focus of SGLT2 inhibitors treatment. Pharmacol Res. (2022) 176:106062. 10.1016/j.phrs.2022.106062 35017046

[181] Nandave M. Role of ACE inhibitors and angiotensin receptor blockers in acute heart failure. In: Nandave M, editor. Angiotensin-converting Enzyme Inhibitors vs Angiotensin Receptor Blockers: A Critical Analysis of Antihypertensive Strategies: A Machine-Generated Literature Overview. Singapore: Springer Nature Singapore (2024). p. 277–327.

[182] Greenberg B. Angiotensin receptor-neprilysin inhibition (ARNI) in heart failure. Int J Heart Fail. (2020) 2:73–90. 10.36628/ijhf.2020.0002 36263291 PMC9536660

[183] de Oliveira MT Jr. Baptista R Chavez-Leal SA Bonatto MG. Heart failure management with beta-blockers: can we do better? Curr Med Res Opin. (2024) 40:43–54. 10.1080/03007995.2024.2318002 38597068

[184] João P F Rossello X Eschalier R McMurray John JV Pocock S Girerd N MRAs in elderly HF patients. JACC Heart Fail. (2019) 7:1012–21. 10.1016/j.jchf.2019.08.017 31779922

[185] Crispino SP Segreti A Nafisio V Valente D Crisci F Ferro A The role of SGLT2-inhibitors across all stages of heart failure and mechanisms of early clinical benefit: from prevention to advanced heart failure. Biomedicines. (2025) 13:608. 10.3390/biomedicines13030608 40149587 PMC11940307

[186] Magdy JS McVeigh J Indraratna P. Diuretics in the management of chronic heart failure: when and how. Aust Prescr. (2022) 45:200–4. 10.18773/austprescr.2022.069 36479331 PMC9722345

[187] Parakh N Chaturvedi V Kurian S Tyagi S. Effect of ivabradine vs atenolol on heart rate and effort tolerance in patients with mild to moderate mitral stenosis and normal sinus rhythm. J Card Fail. (2012) 18:282–8. 10.1016/j.cardfail.2012.01.001 22464768

[188] Armstrong Paul W Roessig L Patel Mahesh J Anstrom Kevin J Butler J Voors Adriaan A A multicenter, randomized, double-blind, placebo-controlled trial of the efficacy and safety of the oral soluble guanylate cyclase stimulator. JACC Heart Fail. (2018) 6:96–104. 10.1016/j.jchf.2017.08.013 29032136

[189] John R T Diaz R Felker GM McMurray John JV Metra M Solomon Scott D Omecamtiv mecarbil in chronic heart failure with reduced ejection fraction. JACC Heart Fail. (2020) 8:329–40. 10.1016/j.jchf.2019.12.001 32035892

[190] Wang W Gao Y Chen Y Cheng M Sang Y Wei L TGF-beta inhibitors: the future for prevention and treatment of liver fibrosis? Front Immunol. (2025) 16:1583616. 10.3389/fimmu.2025.1583616 40655154 PMC12245701

[191] Jiang Y Qiang Z Liu Y Zhu L Xiao L Du Z Diverse functions of NLRP3 inflammasome in PANoptosis and diseases. Cell Death Discov. (2025) 11:389. 10.1038/s41420-025-02689-1 40830103 PMC12365032

[192] Estep JD Salah HM Kapadia SR Burkhoff D Lala A Butler J HFSA scientific statement: update on device based therapies in heart failure. J Card Fail. (2024) 30:1472–88. 10.1016/j.cardfail.2024.07.007 39261158

[193] Nguyen AH Hurwitz M Abraham J Blumer V Flanagan MC Garan AR Medical management and device-based therapies in chronic heart failure. J Soc Cardiovasc Angiogr Interv. (2023) 2:101206. 10.1016/j.jscai.2023.101206 39131076 PMC11308856

[194] Salah HM Fudim M Burkhoff D. Device interventions for heart failure. JACC Heart Fail. (2023) 11:1039–54. 10.1016/j.jchf.2023.07.002 37611987

[195] Debska-Kozlowska A Ksiazczyk M Warchol I Lubinski A. Clinical usefulness of N-terminal prohormone of brain natriuretic peptide and high sensitivity troponin T in patients with heart failure undergoing cardiac resynchronization therapy. Curr Pharm Des. (2019) 25:1671–8. 10.2174/1381612825666190621155718 31223080

[196] Philippon F. Cardiac resynchronization therapy: device-based medicine for heart failure. J Card Surg. (2004) 19:270–4. 10.1111/j.0886-0440.2004.04081.x 15151661

[197] Sun S Joglar JA. Cardiac resynchronization therapy: prospect for long-lasting heart failure remission. J Investig Med. (2011) 59:887–92. 10.2310/JIM.0b013e3182186320 21441824

[198] Suleiman M Goldenberg I Samniah N Rosso R Marai I Pekar A Outcome of patients with advanced heart failure who receive device-based therapy for primary prevention of sudden cardiac death: insights from the Israeli ICD registry. Pacing Clin Electrophysiol. (2015) 38:738–45. 10.1111/pace.12627 25754272

[199] Belkin MN Upadhyay GA. Does cardiac resynchronization therapy benefit patients with non-left bundle branch block prolonged QRS patterns? Curr Cardiol Rep. (2017) 19:125. 10.1007/s11886-017-0929-8 29064041

[200] Rowe MK Kaye GC. Advances in atrioventricular and interventricular optimization of cardiac resynchronization therapy—what’s the gold standard? Expert Rev Cardiovasc Ther. (2018) 16:183–96. 10.1080/14779072.2018.1427582 29338475

[201] Lunati M Magenta G Cattafi G Moreo A Falaschi G Contardi D Clinical relevance of systematic CRT device optimization. J Atr Fibrillation. (2014) 7:1077. 10.4022/jafib.1077 27957096 PMC5135253

[202] Ueda N Noda T Ishibashi K Nakajima K Kataoka N Kamakura T Efficacy of a device-based continuous optimization algorithm for patients with cardiac resynchronization therapy. Circ J. (2019) 84:18–25. 10.1253/circj.CJ-19-0691 31656236

[203] Gasparini M Regoli F Galimberti P Ceriotti C Cappelleri A. Cardiac resynchronization therapy in heart failure patients with atrial fibrillation. Europace. (2009) 11(5):v82–86. 10.1093/europace/eup273 19861396 PMC2768583

[204] Zhang Q Chan YS Liang YJ Fang F Lam YY Chan CP Comparison of left ventricular reverse remodeling induced by cardiac contractility modulation and cardiac resynchronization therapy in heart failure patients with different QRS durations. Int J Cardiol. (2013) 167:889–93. 10.1016/j.ijcard.2012.01.066 22330007

[205] Kramer DB Reynolds MR Mitchell SL. Resynchronization: considering device-based cardiac therapy in older adults. J Am Geriatr Soc. (2013) 61:615–21. 10.1111/jgs.12174 23581915 PMC3628731

[206] Suleiman M Goldenberg I Haim M Schliamser JE Boulos M Ilan M Clinical characteristics and outcomes of elderly patients treated with an implantable cardioverter-defibrillator or cardiac resynchronization therapy in a real-world setting: data from the Israeli ICD registry. Heart Rhythm. (2014) 11:435–41. 10.1016/j.hrthm.2013.12.003 24315966

[207] Lewis GF Gold MR. Developments in cardiac resynchronisation therapy. Arrhythm Electrophysiol Rev. (2015) 4(2):122–8. 10.15420/aer.2015.04.02.122 26835113 PMC4711555

[208] Goldberger Z Lampert R. Implantable cardioverter-DefibrillatorsExpanding indications and technologies. JAMA. (2006) 295:809–18. 10.1001/jama.295.7.809 16478904

[209] Sanchez-Enrique C Jorde UP Gonzalez-Costello J. Heart transplant and mechanical circulatory support in patients with advanced heart failure. Rev Esp Cardiol (Engl Ed). (2017) 70:371–81. 10.1016/j.rec.2016.12.036 28188009

[210] Sharif ZI Galand V Hucker WJ Singh JP. Evolving cardiac electrical therapies for advanced heart failure patients. Circ Arrhythm Electrophysiol. (2021) 14:e009668. 10.1161/CIRCEP.120.009668 33858178

[211] Bazoukis G Saplaouras A Efthymiou P Yiannikourides A Liu T Letsas KP Cardiac contractility modulation in patients with heart failure—a review of the literature. Heart Fail Rev. (2024) 29:689–705. 10.1007/s10741-024-10390-1 38393423

[212] Berry N Mauri L Feldman T Komtebedde J van Veldhuisen DJ Solomon SD Transcatheter InterAtrial shunt device for the treatment of heart failure: rationale and design of the pivotal randomized trial to REDUCE elevated left atrial pressure in patients with heart failure II (REDUCE LAP-HF II). Am Heart J. (2020) 226:222–31. 10.1016/j.ahj.2019.10.015 32629295

[213] Tom SK Kalra K Perdoncin E Tully A Devireddy CM Inci E Transcatheter treatment options for functional mitral regurgitation: which device for which patients? Interv Cardiol. (2024) 19:e10. 10.15420/icr.2021.29 39081829 PMC11287627

[214] Ravassa S Lopez B Treibel TA San Jose G Losada-Fuentenebro B Tapia L Cardiac fibrosis in heart failure: focus on non-invasive diagnosis and emerging therapeutic strategies. Mol Aspects Med. (2023) 93:101194. 10.1016/j.mam.2023.101194 37384998

[215] Gallagher MJ McCullough PA. The emerging role of natriuretic peptides in the diagnosis and treatment of decompensated heart failure. Curr Heart Fail Rep. (2004) 1:129–35. 10.1007/s11897-004-0022-7 16036036

[216] Moayedi Y Ross HJ. Advances in heart failure: a review of biomarkers, emerging pharmacological therapies, durable mechanical support and telemonitoring. Clin Sci. (2017) 131:553–66. 10.1042/CS20160196 28302916

[217] Neuen BL Vaduganathan M Claggett BL Beldhuis I Myhre P Desai AS Natriuretic peptides, kidney function, and clinical outcomes in heart failure with preserved ejection fraction. JACC Heart Fail. (2025) 13:28–39. 10.1016/j.jchf.2024.08.009 39365237

[218] Salah K Stienen S Pinto YM Eurlings LW Metra M Bayes-Genis A Prognosis and NT-proBNP in heart failure patients with preserved versus reduced ejection fraction. Heart. (2019) 105:1182–9. 10.1136/heartjnl-2018-314173 30962192 PMC6662953

[219] Tsutsui H Albert NM Coats AJS Anker SD Bayes-Genis A Butler J Natriuretic peptides: role in the diagnosis and management of heart failure: a scientific statement from the heart failure association of the European society of cardiology, heart failure society of America and Japanese heart failure society. J Card Fail. (2023) 29:787–804. 10.1016/j.cardfail.2023.02.009 37117140

[220] Packer M Anker SD Butler J Filippatos G Ferreira JP Pocock SJ Effect of empagliflozin on the clinical stability of patients with heart failure and a reduced ejection fraction: the EMPEROR-reduced trial. Circulation. (2021) 143:326–36. 10.1161/CIRCULATIONAHA.120.051783 33081531 PMC7834905

[221] Shi Y Zhao L Wang J Liu X Bai Y Cong H Empagliflozin protects against heart failure with preserved ejection fraction partly by inhibiting the senescence-associated STAT1-STING axis. Cardiovasc Diabetol. (2024) 23:269. 10.1186/s12933-024-02366-0 39044275 PMC11267814

[222] Tuttolomondo A Simonetta I Daidone M Mogavero A Ortello A Pinto A. Metabolic and vascular effect of the Mediterranean diet. Int J Mol Sci. (2019) 20:4716. 10.3390/ijms20194716 31547615 PMC6801699

[223] Basu R Poglitsch M Yogasundaram H Thomas J Rowe BH Oudit GY. Roles of angiotensin peptides and recombinant human ACE2 in heart failure. J Am Coll Cardiol. (2017) 69:805–19. 10.1016/j.jacc.2016.11.064 28209222

[224] He X Du T Long T Liao X Dong Y Huang ZP. Signaling cascades in the failing heart and emerging therapeutic strategies. Signal Transduct Target Ther. (2022) 7:134. 10.1038/s41392-022-00972-6 35461308 PMC9035186

[225] deFilippi CR Shah P Shah SJ Alemayehu W Lam CSP Butler J Proteomics identify clinical phenotypes and predict functional outcomes in heart failure with preserved ejection fraction: insights from VITALITY-HFpEF. Circ Heart Fail. (2024) 17:e011792. 10.1161/CIRCHEARTFAILURE.124.011792 39206547

[226] Georgiopoulou VV Tang WHW Giamouzis G Li S Deka A Dunbar SB Renal biomarkers and outcomes in outpatients with heart failure: the Atlanta cardiomyopathy consortium. Int J Cardiol. (2016) 218:136–43. 10.1016/j.ijcard.2016.05.041 27232925

[227] Nagai T Nakao M Anzai T. Risk stratification towards precision medicine in heart failure—current progress and future perspectives. Circ J. (2021) 85:576–83. 10.1253/circj.CJ-20-1299 33658445

[228] Ni W Jiang R Xu D Zhu J Chen J Lin Y Association between insulin resistance indices and outcomes in patients with heart failure with preserved ejection fraction. Cardiovasc Diabetol. (2025) 24:32. 10.1186/s12933-025-02595-x 39844150 PMC11755915

[229] Ezekowitz JA Colin-Ramirez E Ross H Escobedo J Macdonald P Troughton R Reduction of dietary sodium to less than 100 mmol in heart failure (SODIUM-HF): an international, open-label, randomised, controlled trial. Lancet. (2022) 399:1391–400. 10.1016/S0140-6736(22)00369-5 35381194

[230] Kimmoun A Cotter G Davison B Takagi K Addad F Celutkiene J Safety, tolerability and efficacy of rapid optimization, helped by NT-proBNP and GDF-15, of heart failure therapies (STRONG-HF): rationale and design for a multicentre, randomized, parallel-group study. Eur J Heart Fail. (2019) 21:1459–67. 10.1002/ejhf.1575 31423712

[231] Davison BA Abbate A Cotter G Pascual-Figal D Van Tassell B Villota JN Effects of anti-inflammatory therapy in acute heart failure: a systematic review and meta-analysis. Heart Fail Rev. (2025) 30:575–87. 10.1007/s10741-025-10491-5 39939545

[232] Hirano KI Okamura S Sugimura K Miyauchi H Nakano Y Nochioka K Long-term survival and durable recovery of heart failure in patients with triglyceride deposit cardiomyovasculopathy treated with tricaprin. Nat Cardiovasc Res. (2025) 4:266–74. 10.1038/s44161-025-00611-7 39948308

[233] Figueiral M Paldino A Fazzini L Pereira NL. Genetic biomarkers in heart failure: from gene panels to polygenic risk scores. Curr Heart Fail Rep. (2024) 21:554–69. 10.1007/s11897-024-00687-5 39405019

[234] von Lueder TG Kotecha D Atar D Hopper I. Neurohormonal blockade in heart failure. Card Fail Rev. (2017) 3:19–24. 10.15420/cfr.2016:22:2 28785471 PMC5494151

[235] Puniya BL. Artificial-intelligence-driven innovations in mechanistic computational modeling and digital twins for biomedical applications. J Mol Biol. (2025) 437:169181. 10.1016/j.jmb.2025.169181 40316010

[236] Lin M Guo J Gu Z Tang W Tao H You S Machine learning and multi-omics integration: advancing cardiovascular translational research and clinical practice. J Transl Med. (2025) 23:388. 10.1186/s12967-025-06425-2 40176068 PMC11966820

[237] Bastos JM Colaço B Baptista R Gavina C Vitorino R. Innovations in heart failure management: the role of cutting-edge biomarkers and multi-omics integration. J Mol Cell Cardiol Plus. (2025) 11:100290. 10.1016/j.jmccpl.2025.100290 40129519 PMC11930597

[238] Ben-Eltriki M Rafiq A Paul A Prabhu D Afolabi MOS Baslhaw R Adaptive designs in clinical trials: a systematic review-part I. BMC Med Res Methodol. (2024) 24:229. 10.1186/s12874-024-02272-9 39367313 PMC11451232

